# An Aggregation‐Induced Polymerization Poly(Disulfide)‐Drug Nanoplatform for Autoimmune Uveitis Therapy via Inhibiting the cGAS‐STING Pathway

**DOI:** 10.1002/advs.76813

**Published:** 2026-07-29

**Authors:** Yuelan Wu, Wenbo Geng, Qinjin Dai, Wanyun Zhang, Yuxian Lai, Pei Zhang, Chunjiang Zhou, Yinuo Wang, Qingfeng Cao, Xiang Luo, Yujie Lai, Changwei Huang, Peizeng Yang

**Affiliations:** ^1^ Ophthalmology Medical Center, The First Affiliated Hospital of Chongqing Medical University Chongqing Key Laboratory For the Prevention and Treatment of Major Blinding Eye Diseases Chongqing Branch (Municipality Division) of National Clinical Research Centre For Ocular Diseases Chongqing China; ^2^ Department of Ophthalmology Joint Research Laboratory For Ocular Immunology and Retinal Injury Repair The First Affiliated Hospital of Zhengzhou University Henan International Henan Province Eye Hospital Zhengzhou China

**Keywords:** autoimmune uveitis, cell‐free DNA, cGAS‐STING pathway, drug delivery, high permeability

## Abstract

Autoimmune uveitis is a sight‐threatening inflammatory disease in which the cGAS‐STING signaling pathway exacerbates inflammation by recognizing excessive cell‐free DNA (cfDNA). However, conventional cfDNA scavengers have limited blood‐retinal barrier penetration, and monotherapies cannot control the complex inflammatory network. Herein, we developed a cationic poly(disulfide)‐drug nanoplatform (LA/DexP), which self‐assembled via salt bridge interactions and aggregation‐induced polymerization between a guanidyl‐functionalized lipoic acid derivative (LA) and dexamethasone phosphate (DexP). This platform is designed to synergistically inhibit the cGAS‐STING pathway and achieve efficient intraocular drug delivery. LA/DexP functioned as an efficient scavenger of cfDNA through electrostatic interaction, thereby inhibiting cGAS‐STING overactivation. Meanwhile, it leveraged thiol‐disulfide exchange to enhance blood‐retinal barrier penetration, achieving a 5.09‐fold higher apparent permeability coefficient than free DexP, and exhibited ROS‐responsive drug release. In an experimental autoimmune uveitis mouse model, LA/DexP treatment significantly reduced cfDNA levels and inhibited cGAS‐STING signaling. It also downregulated pro‐inflammatory cytokine expression, promoted macrophage polarization from the pro‐inflammatory M1 to the anti‐inflammatory M2 phenotype, and rebalanced Th1/Th17‐Treg cell subsets. These combined effects effectively inhibited uveitis severity. In summary, this study establishes a highly barrier‐permeable nanoplatform that synergistically integrates drug delivery with cGAS‐STING pathway inhibition, offering a promising therapeutic strategy for autoimmune uveitis.

## Graphical abstract

A cationic poly(disulfide)‐drug nanoplatform (LA/DexP) was developed to treat experimental autoimmune uveitis (EAU). With potent blood‐retinal barrier penetrability, LA/DexP releases DSP in response to high ROS and scavenges cfDNA to inhibit the cGAS‐STING signaling pathway. These synergistic effects downregulate pro‐inflammatory mediators, induce M2 macrophage polarization, restore T cell balance, and ultimately ameliorate ocular inflammation in EAU mice.

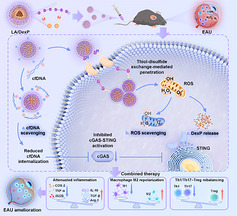

## 1 Introduction

Autoimmune uveitis (AU) is a chronic, recurrent intraocular inflammatory disease that can lead to severe visual impairment or even blindness [1, 2]. In developing countries, approximately 25% of severe vision impairment cases are attributable to AU [3]. Although its exact etiology remains to be elucidated, the pathological core involves persistent aberrant immune attacks on ocular tissues, creating a pro‐inflammatory microenvironment characterized by blood‐retinal barrier (BRB) disruption, inflammatory cell infiltration, reactive oxygen species (ROS) burst, and progressive retinal damage [4, 5, 6]. The cGAS‐STING signaling pathway, a key component of innate immunity, has emerged as a critical hub for amplifying inflammatory responses in autoimmune diseases including AU [7]. Cell damage or death releases large amounts of cell‐free DNA (cfDNA) into the extracellular space or circulation [8, 9]. As a critical damage‐associated molecular pattern, cfDNA activates the cGAS‐STING cascade, which promotes inflammatory cytokine release and macrophage polarization toward the pro‐inflammatory M1 phenotype, thereby aggravating inflammation [10, 11, 12]. Elevated serum cfDNA levels and aberrant retinal activation of the cGAS‐STING pathway have been consistently observed in experimental autoimmune uveitis (EAU) animal models by multiple research groups, including ours [13, 14, 15]. Therefore, scavenging cfDNA and inhibiting the cGAS‐STING pathway holds great promise for controlling chronic inflammation of AU.

Cationic nanomaterials have shown therapeutic potential as cfDNA scavengers and pathway modulators in various inflammatory diseases [9, 16]. For instance, polyethyleneimine‐coated mesoporous polydopamine nanoparticles effectively adsorb cfDNA via electrostatic interactions and inhibit cGAS recognition, alleviating joint damage in rheumatoid arthritis mouse models [17]. However, these materials often face limitations such as poor serum stability and potential cytotoxicity [18]. Critically, inhibition of a single inflammatory axis is often insufficient to achieve sustained disease remission. Glucocorticoids, such as dexamethasone sodium phosphate (DexP), provide broad‐spectrum anti‐inflammatory effects that could complement cGAS‐STING inhibition. Nonetheless, their long‐term application may lead to severe systemic side effects, such as infection, metabolic disorders, and osteoporosis [19, 20]. Notably, the tight BRB severely restricts intraocular delivery of both biomaterials and conventional drugs, leading to low ocular bioavailability [21]. Therefore, there is an urgent need to develop a low‐toxicity, high‐permeability co‐delivery system that integrates cGAS‐STING pathway inhibition with conventional anti‐inflammatory therapy for the comprehensive alleviation of uveitis.

Stimuli‐responsive materials based on dynamic covalent chemistry offer new opportunities for intraocular drug delivery. Poly(disulfide)s derived from ring‐opening polymerization of the endogenous molecule lipoic acid exhibit excellent biocompatibility and biodegradability [22, 23]. Their dynamic disulfide bonds can undergo specific thiol‐disulfide exchange with thiol‐rich membrane proteins, driving thiol‐mediated uptake (TMU) and enabling nanoparticles to overcome tight physiological barriers [24, 25]. TMU‐based delivery systems have been shown to significantly enhance target tissue accumulation [26]. Moreover, disulfide bonds are sensitive to the redox environment and can be selectively cleaved by elevated ROS in the pathological microenvironment [27]. This feature facilitates the controlled release of loaded drugs, while simultaneously consuming ROS to alleviate oxidative stress. Furthermore, the carboxyl groups on poly(disulfide)s side chains provide convenient sites for functional modification [28]. This allows for the conjugation of cationic units such as guanidinium groups, equipping the carrier with the ability to capture cfDNA.

**SCHEME 1:**
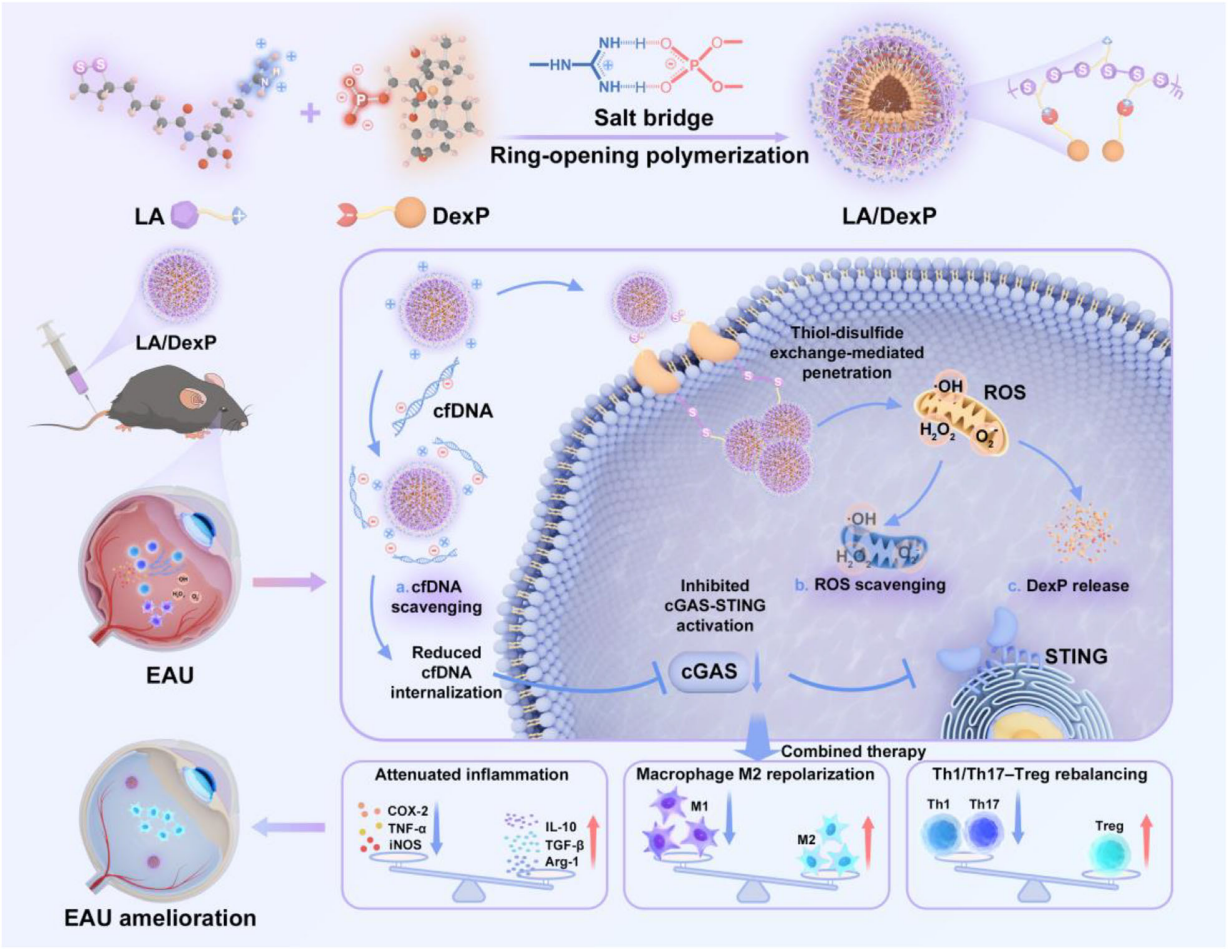
Therapeutic mechanism of LA/DexP in EAU. LA/DexP crosses the blood‐retinal barrier via thiol‐disulfide exchange and accumulates in the retina. At inflammatory sites, it releases DexP via ROS‐responsive cleavage, scavenges ROS, and electrostatically neutralizes cfDNA to inhibit the cGAS‐STING pathway. This synergistic action suppresses inflammatory cytokines, promotes M2 macrophage polarization and T‐cell balance, thereby alleviating EAU.

Motivated by the above advantages of Poly(disulfide)s, we designed a barrier‐permeable cationic poly(disulfide)‐drug nanoplatform, termed LA/DexP, for synergistic cGAS‐STING pathway inhibition and DexP delivery in AU therapy (Scheme 1). This platform was formed via salt‐bridge‐driven aggregation‐induced ring‐opening polymerization and self‐assembly between guanidyl‐functionalized lipoic acid derivative (LA) and DexP. We demonstrated that LA/DexP exhibits excellent biocompatibility and high barrier permeability, enabling efficient accumulation in retinal tissues. It effectively bound and scavenged cfDNA, reduced its cellular internalization, and suppressed cGAS‐STING pathway activation in macrophages. Moreover, it demonstrated ROS‐responsive DexP release and ROS‐scavenging capacity, exerting both anti‐inflammatory and antioxidant effects. In an EAU mouse model, LA/DexP significantly alleviated retinal inflammatory infiltration and tissue damage, while alleviating oxidative stress and restoring blood‐retinal barrier integrity. Further studies revealed that LA/DexP reduced pro‐inflammatory cytokines, promoted macrophage polarization toward the anti‐inflammatory M2 phenotype, and restored immune balance between Th1/Th17 and Treg cells. In summary, LA/DexP represents a synergistic nano‐strategy, demonstrating considerable therapeutic potential for AU.

## 2 Results and Discussion

### 2.1 Elevated cfDNA Triggers cGAS‐STING Pathway Activation in AU

**FIGURE 1:**
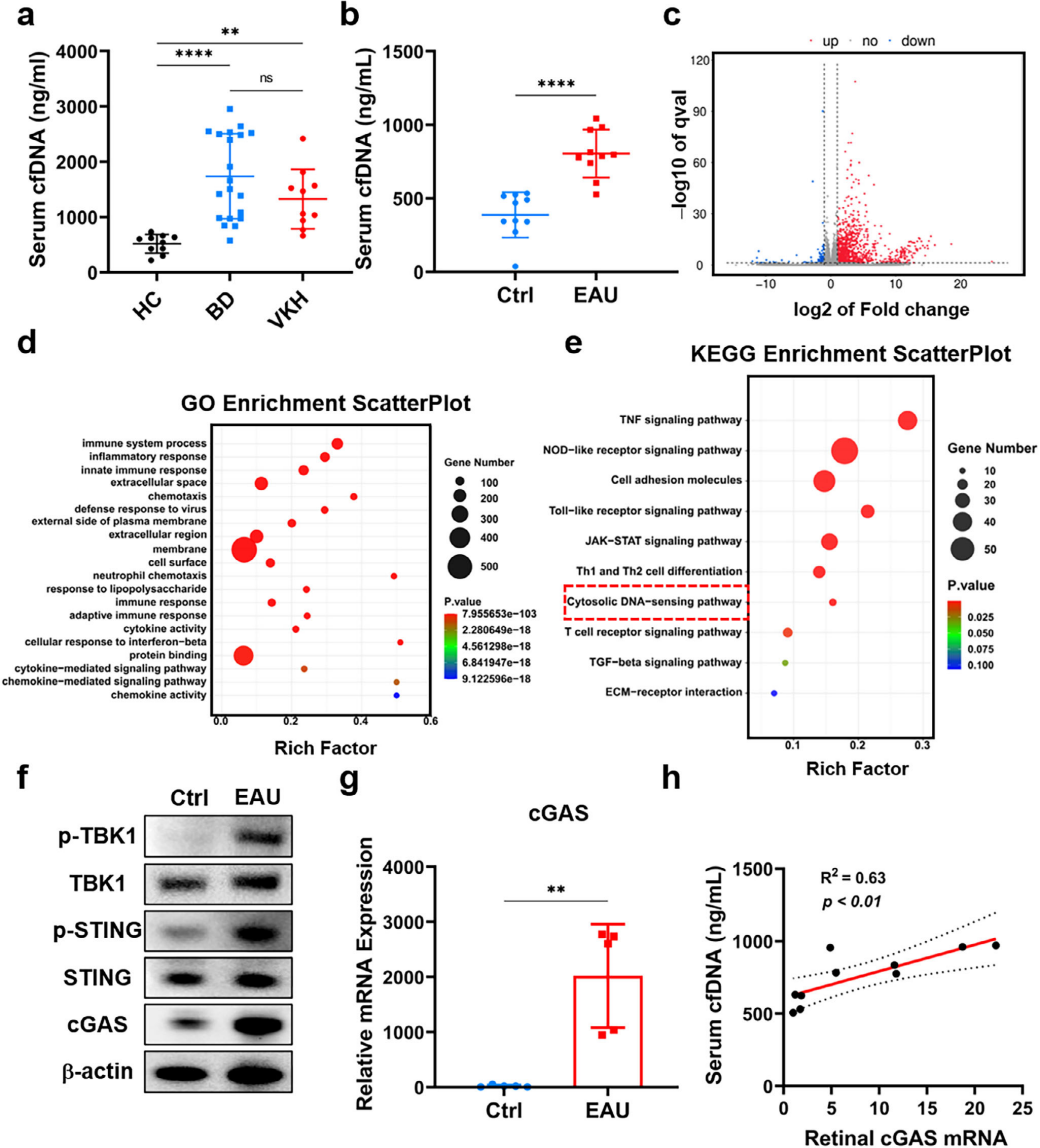
Elevated cfDNA and cGAS‐STING pathway activation are observed in AU. (a) Serum cfDNA levels in healthy controls (HC, n = 10) and active AU patients (BD and VKH, n = 30). (b) Serum cfDNA levels in normal controls and EAU mice (n = 10). (c) Volcano plot of DEGs in retinal tissues from normal controls and EAU mice, based on RNA‐seq analysis (|log_2_ fold change| > 1, adjusted *p <* 0.05). (d) GO functional enrichment analysis of DEGs. (e) KEGG pathway enrichment analysis of DEGs. (f) Representative western blot images of key cGAS‐STING pathway proteins (cGAS, STING, p‐STING, TBK1, p‐TBK1) in retinal tissues from normal controls and EAU mice (n = 5). (g) qRT‐PCR analysis of cGAS mRNA expression in retinal tissue from normal controls and EAU mice (n = 5). (h) Pearson correlation analysis of retinal cGAS mRNA expression and serum cfDNA levels in EAU mice (n = 10). Data are presented as mean ± SD; **p < 0.01; ****p < 0.0001.

The cGAS‐STING signaling pathway serves as a central axis linking cytosolic DNA sensing to innate immune responses and plays a critical role in inflammatory regulation [7, 29]. As a classic ligand, cfDNA has been shown to trigger this pathway [30]. However, whether cfDNA contributes to AU pathogenesis through cGAS‐STING activation remains unclear. In China, Behcet's disease (BD) and Vogt‐Koyanagi‐Harada (VKH) syndrome are the two most common subtypes of AU [31]. To explore the specific role of cfDNA, we measured serum cfDNA levels in 30 patients with active disease (20 BD, 10 VKH) and 10 healthy volunteers. As shown in Figure 1a, serum cfDNA levels were significantly higher in active AU patients compared to healthy controls, with no significant difference observed between the BD and VKH subgroups. Moreover, cfDNA levels showed no correlation with patient gender or age (Figure S1). Using an interphotoreceptor retinoid‐binding protein (IRBP)‐induced EAU mouse model, we further confirmed that serum cfDNA levels were also significantly elevated at the peak of disease (Figure 1b). These results indicate that elevated cfDNA is a common feature of AU.

To determine whether elevated cfDNA activates the cGAS‐STING pathway, we performed RNA sequencing on retinal tissues from control and EAU mice. A total of 1,228 differentially expressed genes (DEGs) were identified, with 1,088 upregulated and 140 downregulated (Figure 1c). Gene Ontology (GO) enrichment analysis revealed that these DEGs were mainly enriched in the biological functional categories including “immune system process”, “inflammatory response” and “innate immune response” (Figure 1d), suggesting that the retina was in a state of intense immune activation. Notably, KEGG pathway enrichment analysis clearly pointed out the activation of the “cytosolic DNA sensing pathway” (Figure 1e). Molecular validation demonstrated that both protein and mRNA levels of cGAS were significantly elevated in EAU retinal tissues, accompanied by increased phosphorylation of STING (p‐STING) and TANK‐binding kinase 1 (p‐TBK1) (Figure 1f,g; Figure S2a). Correlation analysis revealed a significant positive correlation between retinal cGAS mRNA levels and serum cfDNA concentrations (R^2^ = 0.63, p < 0.01; Figure 1h). In vitro experiments further confirmed that stimulation of RAW264.7 cells with the cfDNA mimetic CpG oligodeoxynucleotide (CpG) significantly upregulated both protein and mRNA levels of cGAS and increased the levels of p‐STING and p‐TBK1 (Figure S2b, c). Collectively, these results demonstrate that elevated cfDNA triggers the activation of the cGAS‐STING pathway in AU, providing a rationale for developing nanotherapies aimed at scavenging cfDNA and inhibiting this pathway.

### 2.2 Synthesis and Characterization of LA/DexP

**FIGURE 2:**
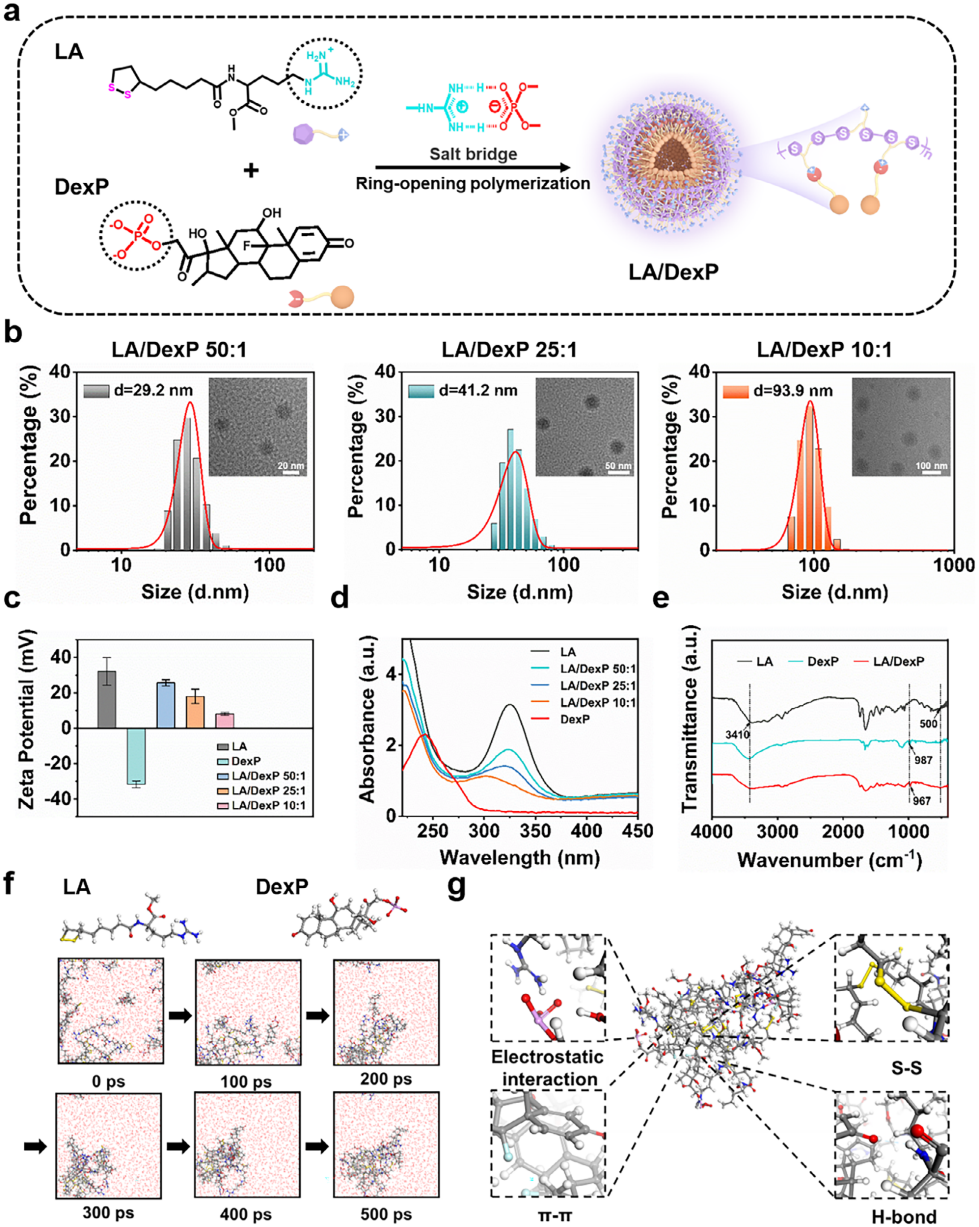
Synthesis and characterization of LA/DexP. (a) Schematic illustration of the co‐assembly process forming LA/DexP nanoparticles. (b) Hydrodynamic size distribution of LA/DexP nanoparticles prepared at different LA:DexP molar ratios (50:1, 25:1, and 10:1). Inset: Representative TEM image of the nanoparticles. (c) Zeta potential of LA/DexP nanoparticles at different molar ratios. (d) UV–vis absorption spectra of LA, DexP and LA/DexP. (e) FTIR spectra of LA, DexP, and LA/DexP. (f) Molecular dynamics simulation snapshots of LA/DexP co‐assembly. (g) Key intermolecular interactions stabilizing the LA/DexP nanostructure.

Leveraging the dynamic covalent chemistry and functional tunability of poly(disulfide)s, we constructed a multifunctional poly(disulfide)‐drug nanoplatform. First, a cationic monomer LA was synthesized via guanidine functionalization of lipoic acid following a reported method [32]. Its structure was verified by ^1^H nuclear magnetic resonance (^1^H NMR) spectroscopy (Figure S3) and matrix‐assisted laser desorption/ionization time‐of‐flight mass spectrometry (MALDI‐TOF MS) measured a molecular mass of 377.17 g/mol, matching the theoretical value (Figure S4). Subsequently, the cationic guanidine groups of LA and the anionic phosphate groups of DexP drove co‐assembly via salt bridges (Figure 2a). During assembly, close intermolecular contact facilitated dynamic disulfide exchange, which triggered ring‐opening polymerization and led to the formation of a crosslinked poly(disulfide) framework [33, 34]. These synergistic interactions stabilize the nanostructure, yielding LA/DexP nanoparticles with a hydrophobic core and a hydrophilic, positively charged surface.

To optimize the formulation, we prepared LA/DexP nanoparticles at different molar ratios (50:1, 25:1, and 10:1). Dynamic light scattering (DLS) analysis revealed hydrodynamic diameters of 29.2, 41.2, and 93.9 nm, respectively (Figure 2b). Transmission electron microscopy (TEM) confirmed the formation of spherical and well‐dispersed nanoparticles with sizes consistent with the DLS results (Figure 2b, inset). Zeta potential analysis showed surface charges of +25.6 mV, +18.0 mV, and +8.1 mV for the corresponding formulations (Figure 2c). Particle size and surface charge are key determinants of the in vivo fate of nanoparticles. Previous studies have shown that nanoparticles in the 10–60 nm range effectively penetrate the BRB and facilitate cellular uptake, whereas particles smaller than 10 nm are rapidly cleared by glomerular filtration [35, 36]. Moreover, a moderately positive surface charge promotes interaction with negatively charged cell membranes and cfDNA, while an excessively high charge may increase potential toxicity [35]. In this experiment, we found that the 10:1 formulation exhibited poor colloidal stability and aggregation, which was caused by increased particle size and low surface charge and may weaken cfDNA binding. The 50:1 formulation, despite having the smallest size, showed the highest surface charge, which may raise toxicity concerns. Considering the balance of size, charge, and colloidal stability, we selected the 25:1 formulation as the optimal candidate for subsequent studies.

To further elucidate the assembly mechanism, we performed a series of spectroscopic analyses. UV‐visible (UV–Vis) spectroscopy revealed that with increasing DexP concentration, the characteristic absorption peak of LA at 330 nm, attributed to its five‐membered ring, gradually decreased in intensity and exhibited a blue shift (Figure 2d), indicating the occurrence of ring‐opening polymerization during co‐assembly. Consistently, the ^1^H NMR spectrum of LA/DexP displayed a characteristic signal at 2.8 ppm corresponding to the disulfide‐bond methylene protons (Figure S5) [37], and MALDI‐TOF MS detected oligomer peaks ranging from dimers to hexamers (Figure S6). Fourier transform infrared (FTIR) analysis provided further evidence, showing broadening of the N─H stretching vibration (3410 cm^−^^1^) and attenuation of the S–S stretching signal (400–500 cm^−^^1^) from LA, along with a red shift of the P─O^−^ vibration peak of DexP from 987 cm^−^^1^ to 967 cm^−^^1^ (Figure 2e). TEM‐based elemental mapping further revealed that the nanoparticles were composed of C, N, O, F, P, and S (Figure S7). To gain deeper insight into the assembly dynamics, we performed molecular dynamics simulations. The results showed that LA and DexP, through electrostatic attraction, could spontaneously assemble from a dispersed state in aqueous solution into compact and stable nanoparticles within approximately 300 ps, accompanied by the formation of S‐S covalent cross‐links (Figure 2f). During this process, the solvent‐accessible surface area decreased by more than 52.1%, while the number of hydrogen bonds gradually increased and eventually stabilized at around 55 (Figure S8), indicating that hydrophobic interactions and hydrogen bonding jointly maintain nanoparticle stability. Key intermolecular interaction analysis further indicated that the assembly was primarily driven by synergistic non‐covalent interactions, including electrostatic interaction, hydrogen bonds, and π–π stacking (Figure 2g). The size and zeta potential of LA/DexP were measured at day 0, 3, 7, 14 and 21 during incubation in water, phosphate‐buffered saline (PBS), or Dulbecco's Modified Eagle Medium (DMEM) containing 10% fetal bovine serum (FBS). Negligible changes in particle size and zeta potential throughout the incubation period demonstrate the favorable colloidal stability of LA/DexP, which makes it suitable for subsequent biological studies (Figure S9). For in vitro and in vivo tracking, the near‐infrared fluorophore Cy5.5 was incorporated into LA/DexP. Confocal microscopy images revealed clear red fluorescence, confirming successful labeling (Figure S10). Taken together, these results collectively demonstrate the successful assembly of LA and DexP into structurally stable nanoparticles.

### 2.3 LA/DexP Exhibits Efficient cfDNA and ROS Scavenging, and ROS‐Responsive Drug Release

**FIGURE 3:**
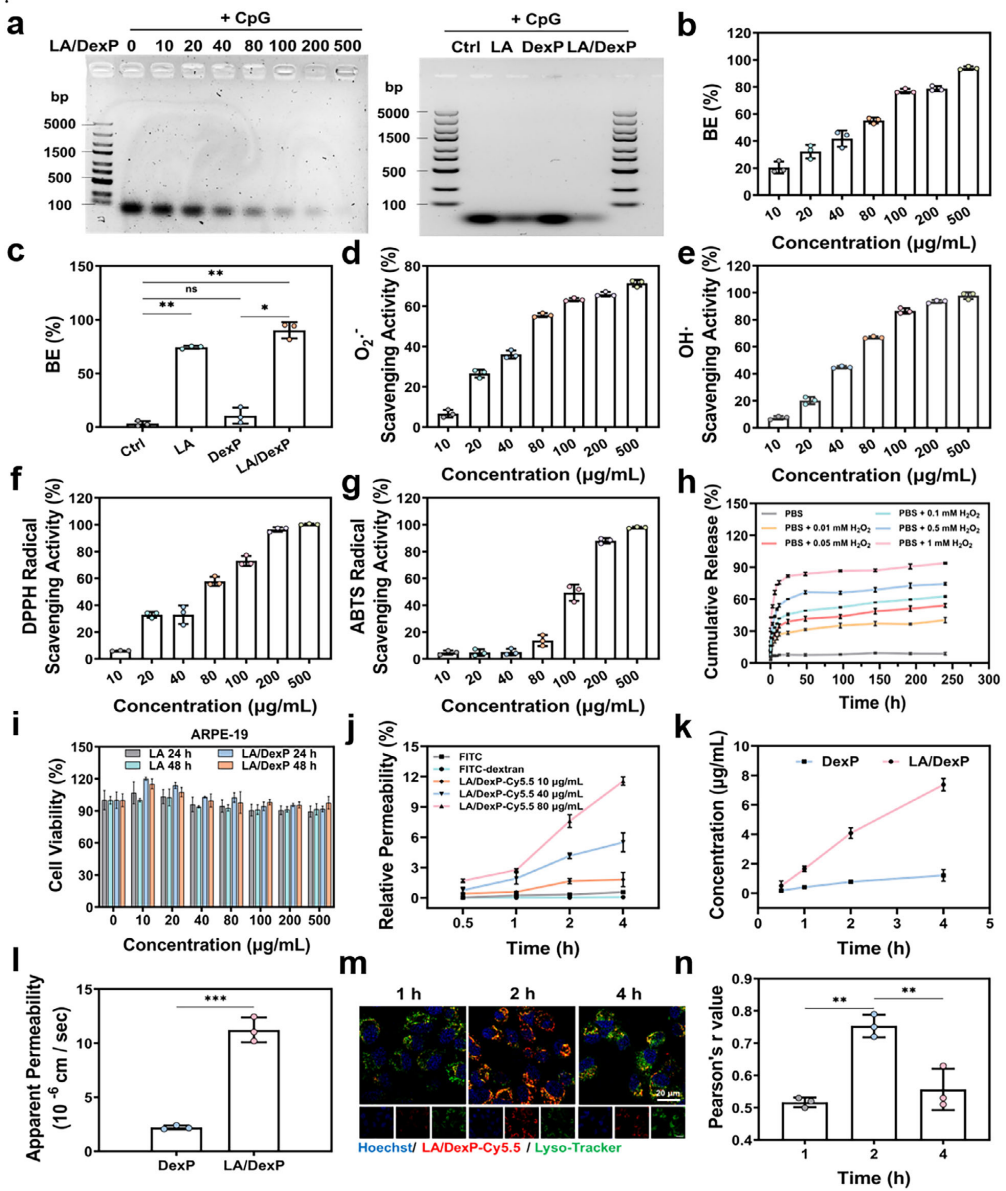
LA/DexP serves as a multifunctional nanoplatform: scavenging ROS/cfDNA, enabling ROS‐responsive drug release, and penetrating the simulated BRB. (a) Agarose gel images of CpG treated with LA/DexP at different concentrations (left) and with PBS, LA, DexP, and LA/DexP (right). (b) DNA binding efficiency of LA/DexP at different concentrations using CpG as the substrate (n = 3). (c) DNA binding efficiency of different treatment groups (PBS, LA, DexP, and LA/DexP, n = 3). (d–g) ROS scavenging capacity. Scavenging rates for (d) O_2_•^−^, (e) •OH, (f) DPPH•, and (g) ABTS•^+^ at different LA/DexP concentrations (n = 3). (h) H_2_O_2_‐responsive release profiles of DexP from LA/DexP nanoparticles in PBS (pH 7.4) at 37°C under different H_2_O_2_ concentrations (0, 0.01, 0.05, 0.1, 0.5, 1.0 mM). (i) Cell viability of ARPE‐19 cells treated with LA or LA/DexP (0–500 µg/mL) for 24 and 48 h using the CCK‐8 assay. (j) Relative permeability of LA/DexP‐Cy5.5 over time at varying concentrations in an ARPE‐19 Transwell model. (k) HPLC quantification of DexP in the bottom chamber over time following treatment with free DexP or LA/DexP nanoparticles. (l) Papp of DexP and LA/DexP at 2 h (n = 3). (m) Representative confocal microscopy images showing the cellular uptake and lysosomal colocalization of LA/DexP‐Cy5.5 at different time points. Scale bar: 20 µm. (n) Quantitative analysis of the colocalization using Pearson's correlation coefficient (n = 3). Data are presented as mean ± SD; *p < 0.05; **p < 0.01; ***p < 0.001.

After confirming the successful synthesis of LA/DexP, we comprehensively evaluated its functional properties in vitro. Agarose gel electrophoresis revealed that LA/DexP efficiently bound and scavenged model DNA including CpG and calf thymus DNA (ctDNA) in a dose‐dependent manner, with performance superior to its individual components (LA or DexP) (Figure 3a; Figure S11). Validation using a broad‐range DNA ladder confirmed its broad‐spectrum scavenging capability, showing a reduction in fragments of 200–12,000 bp at 100–200 µg/mL and their complete elimination at 500 µg/mL (Figure S12). Ethidium bromide competitive binding assays further quantified its high binding efficiency, reaching 94% at 500 µg/mL (Figure 3b,c). It is well established that serum protein adsorption is a major factor leading to rapid clearance and poor tissue accumulation of cationic nanoparticles in vivo [38]. Notably, LA/DexP maintained efficient cfDNA scavenging capability in medium containing 10% FBS, comparable to its performance in PBS (Figure S13a), demonstrating excellent resistance to protein interference. This property stems from the adaptive surface of its dynamic covalent network [39], which effectively reduces nonspecific protein binding, thereby contributing to prolonged in vivo circulation and enhanced tissue accumulation. Furthermore, UV–Vis (Figure S13b) and fluorescence spectroscopy (Figure S13c) revealed significant spectral shifts and fluorescence enhancement upon LA/DexP binding to CpG, indicating a direct interaction between LA/DexP and DNA. Collectively, these results demonstrate that LA/DexP possesses efficient, broad‐spectrum, and stable cfDNA scavenging capabilities in vitro.

Activated immune cells produce abundant ROS, leading to direct tissue damage and secondary cfDNA release via mitochondrial disruption, which collectively amplify the inflammatory response [40]. Given the critical pathogenic role of ROS, we evaluated the ROS scavenging capacity of LA/DexP against key reactive species involved in this process. LA/DexP efficiently scavenged superoxide anion (O_2_•^−^), the primary product of NADPH oxidase activation, and hydroxyl radical (•OH), the most destructive species in oxidative damage, in a concentration‐dependent manner (Figure 3d,e). As hydrogen peroxide (H_2_O_2_) is a pivotal intermediate in the ROS cascade [41], we further analyzed its clearance using an H_2_O_2_‐specific fluorescent probe. Both prolonged incubation time and increased LA/DexP concentration enhanced H_2_O_2_ scavenging efficiency, as reflected by decreased fluorescence signals (Figure S14). Furthermore, LA/DexP exhibited broad‐spectrum scavenging activity against the classical stable radical models, including 2,2‐diphenyl‐1‐picrylhydrazyl (DPPH•) and 2,2'‐azino‐bis(3‐ethylbenzothiazoline‐6‐sulfonic acid) (ABTS•^+^), achieving nearly complete elimination at 500 µg/mL (Figures 3f, g). Therefore, LA/DexP demonstrates effective and broad‐spectrum ROS scavenging capabilities.

Based on the redox sensitivity of its poly(disulfide) backbone, we further investigated the ROS‐triggered drug release behavior of LA/DexP. Prior to release studies, we first characterized the drug loading capacity of LA/DexP, which exhibited an encapsulation efficiency of 90.06% and a drug loading content of 8.11% (Figure S15). Although this loading content is relatively low owing to the high carrier‐to‐drug ratio, the improved permeability of LA/DexP increases cellular uptake of DexP, which helps achieve an effective therapeutic concentration. Meanwhile, given the potent anti‐inflammatory activity of DexP at low doses, the reduced payload does not undermine its therapeutic potential. With the drug loading confirmed, we examined the release kinetics under varying H_2_O_2_ concentrations, including low levels (0.01 and 0.05 mM) to mimic mild oxidative stress and higher levels (0.1–1.0 mM) to simulate severe oxidative stress. As shown in Figure 3h, under physiological conditions (PBS, pH 7.4), the release of encapsulated DexP was slow, with cumulative release remaining below 7.79% within 12 h. This indicates that the system possesses good stability under normal conditions, helping to reduce the risk of systemic toxicity from non‐specific drug release. In contrast, upon exposure to H_2_O_2_, the release of DexP was markedly accelerated in a concentration‐dependent manner with cumulative release ranging from 27.14% to 75.85% within 12 h. Notably, even at low H_2_O_2_ concentrations, the system exhibited appreciable release, confirming its sensitivity to mild oxidative stress. This stimulus‐responsive behavior originates from the ROS‐selective cleavage of disulfide bonds, which destabilizes the assembly and triggers burst release. After the initial burst release, the system maintained a sustained‐release profile for up to 240 h, supporting its potential to extend therapeutic duration and reduce dosing frequency. Further structural analysis revealed that after treatment with 1 mM H_2_O_2_ for 12 h, the average nanoparticle size decreased from 43.3 to 23.7 nm (Figure S16a), while the zeta potential increased from +22.8 to +39.6 mV (Figure S16b). TEM images showed local swelling and blurred contours of the particles (Figure S16c), collectively confirming ROS‐triggered structural disassembly of LA/DexP. Consequently, LA/DexP not only exhibits excellent cfDNA and ROS scavenging capabilities but also responds to ROS in a concentration‐dependent manner, enabling on‐demand controlled release of DexP. Owing to this differential ROS response, LA/DexP maintains structural integrity to scavenge ROS during blood circulation yet undergoes gradual degradation within the high‐ROS microenvironment of inflamed retinas [42]. These features establish it as an advanced drug delivery platform suitable for the treatment of uveitis.

### 2.4 LA/DexP Demonstrates Good Cytocompatibility, Trans‐Barrier Penetration, and Thiolmediated‐ Cellular‐ Uptake

The biocompatibility of nanomaterials is a crucial consideration for biomedical applications. Accordingly, we evaluated the in vitro cytotoxicity of LA and LA/DexP. The cell viability of human retinal pigment epithelial cells (ARPE‐19), murine macrophage cells (RAW264.7), and murine fibroblast cells (L‐929) treated with LA or LA/DexP at concentrations ranging from 0 to 500 µg/mL was assessed using the Cell Counting Kit‐8 (CCK‐8) assay. After 24 h or 48 h of incubation, viability remained above 85% in all tested groups (Figure 3i; Figure S17). Calcein‐AM/PI staining showed that most cells treated with LA or LA/DexP maintained viability, intact morphology, and uniform distribution (Figure S18). Together, these data collectively confirm the good cytocompatibility of LA and LA/DexP within the tested concentration range.

The BRB is a highly selective physiological barrier that strictly limits drug transport from the bloodstream into the retina [21, 43]. Overcoming the BRB is therefore essential for effective intraocular drug delivery. To investigate the trans‐barrier permeation behavior of LA/DexP, we constructed an in vitro BRB model using confluent ARPE‐19 monolayers. Transepithelial electrical resistance (TER) readings reached a stable plateau by day 14, and ZO‐1 immunofluorescence staining revealed continuous tight junction signals along intercellular borders (Figure S19). These observations indicated the establishment of a structurally intact barrier. We further verified its functional integrity using small‐molecule sodium fluorescein and large‐molecule FITC‐dextran in a Transwell system. Both tracers showed negligible transmembrane permeation (Figure 3j). In contrast, LA/DexP crossed the monolayer in a time‐dependent (0.5–4 h) and concentration‐dependent (10–80 µg/mL) manner. Importantly, neither TER values nor ZO‐1 staining distribution was altered after 4 h exposure to LA/DexP (Figure S20), confirming that the nanoparticles promoted its own penetration without compromising barrier integrity. Quantitative HPLC analysis showed that the permeated concentration of LA/DexP in the bottom chamber exceeded that of free DexP at all tested time points (Figure 3k). The apparent permeability coefficient (Papp) of LA/DexP was calculated as (11.22 ± 1.14) × 10^−^^6^ cm/s, approximately 5.09‐fold higher than free DexP (Figure 3l). Considering that ARPE‐19 cells develop relatively loose tight junctions and may not fully mimic the high‐resistance native BRB in vivo [44], we further isolated primary RPE cells from C57BL/6J mice to construct a tighter in vitro barrier model. RPE65 immunofluorescence staining at day 3 post‐isolation identified pure primary RPE populations with distinct positive signals (Figure S21a). Continuous TER monitoring over 28 days of culture revealed a gradual rise in transepithelial resistance that stabilized from day 20 onward, indicating full monolayer maturation (Figure S21b). Bright‐field images of cell monolayers cultured for 20 days displayed typical cobblestone morphology with abundant intracellular melanin pigment, demonstrating complete cell confluence (Figure S21c). Meanwhile, ZO‐1 immunofluorescence staining visualized continuous, intact tight junction outlines between neighboring RPE cells (Figure S21d). Both morphological and electrical evidence confirms successful construction of mature primary RPE barriers. Trans‐barrier permeation of free DexP and LA/DexP was quantified via HPLC detection of basolateral DexP accumulation at serial time points (Figure S21e). Consistent with the ARPE‐19 monolayer results, LA/DexP exhibited time‐dependent transport and superior permeability compared with free DexP, with a Papp value of (4.57 ± 0.35) × 10^−^^6^ cm/s, approximately 4.53‐fold higher than that of free DexP (Figure S21f). These results from both RPE monolayer models demonstrate that LA/DexP possesses trans‐barrier penetration capacity.

To elucidate the cellular mechanism underlying the trans‐barrier delivery of LA/DexP, we further tracked its cellular uptake and intracellular trafficking by confocal microscopy. Fluorescence intensity increased markedly from 1 to 2 h, confirming efficient cellular internalization (Figure 3m). At 2 h, nanoparticles showed strong co‐localization with lysosomes (Pearson's r = 0.75). By 4 h, both total intracellular fluorescence and lysosomal co‐localization decreased significantly (Pearson's r = 0.55; Figure 3n), indicating that a portion of the nanoparticles escaped from the lysosomal compartment after uptake. Given that the poly(disulfide) backbone can undergo exchange reactions with cell surface thiols, we hypothesized that this process might mediate the cellular uptake and subsequent escape of LA/DexP. To test this hypothesis, cells were pretreated with 5,5'‐dithiobis‐(2‐nitrobenzoic acid) (DTNB) to block thiol‐disulfide exchange [25]. This pretreatment markedly reduced intracellular Cy5.5 fluorescence at all time points (Figure S22), confirming that LA/DexP uptake depends on accessible cell‐surface thiols. This thiol‐mediated uptake mechanism facilitates nanoparticle internalization and lysosomal escape [45], which helps prevent drug degradation in lysosomes, allowing the drug to reach the cytoplasm in an active form for therapeutic action. In summary, these in vitro cellular data demonstrate the potential of LA/DexP for intraocular drug delivery, as it exhibits good cytocompatibility and achieves trans‐barrier penetration and cellular uptake without compromising BRB integrity.

### 2.5 LA/DexP Inhibits cfDNA‐Induced cGAS‐STING Activation and Reshapes Macrophage Phenotype In Vitro

**FIGURE 4:**
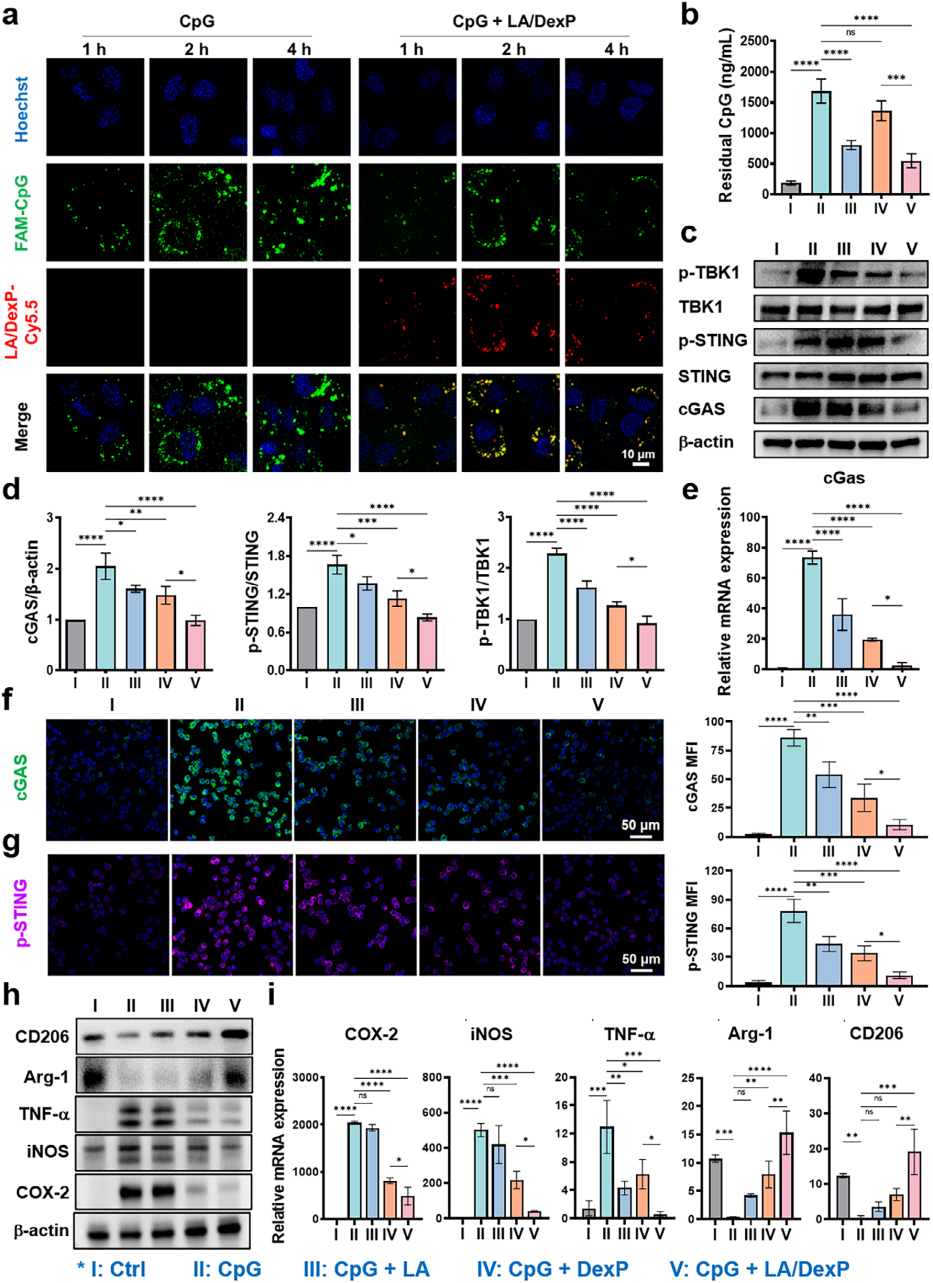
LA/DexP reduces cfDNA internalization and inhibits the cGAS‐STING pathway in vitro. (a) Fluorescence images of RAW264.7 cells treated with FAM‐CpG (green) alone or together with LA/DexP‐Cy5.5 (red). Nuclei were stained with Hoechst (blue). Scale bar: 10 µm. (b) Residual CpG concentration in supernatant after 12 h co‐treatment with CpG and PBS, LA, DexP, or LA/DexP (n = 3). (c, d) Western blot analysis of key cGAS‐STING pathway components under different treatments (Ctrl, CpG, CpG + LA, CpG + DexP, CpG + LA/DexP). (c) Representative blots. (d) Quantitative analysis of protein expression (n = 3). (e) qRT‐PCR analysis of cGAS mRNA expression (n = 3). (f, g) Immunofluorescence staining and quantification of cGAS (f) and STING (g). Nuclei were stained with DAPI (blue). Scale bar: 20 µm (n = 3). (h) Western blot analysis of COX‐2, iNOS, TNF‐α, Arg‐1, and CD206 protein levels, and (i) qRT‐PCR analysis of their corresponding mRNA expression (n = 3). Data are presented as mean ± SD; *p < 0.05; **p < 0.01; ***p < 0.001; ****p < 0.0001.

Although previous studies have confirmed the efficient cfDNA‐binding capacity of LA/DexP nanoparticles in solution, whether they modulate cfDNA‐triggered immune signaling and cellular functions in a complex cellular environment remains unclear. To address this, we first investigated the effect of LA/DexP on cfDNA clearance in RAW264.7 cells co‐incubated with FAM‐CpG and LA/DexP‐Cy5.5 for 4 h. Confocal microscopy revealed that when FAM‐CpG was applied alone, its intracellular green fluorescence intensity gradually increased over time, indicating that CpG was recognized by cells and then internalized (Figure 4a; Figure S23). In contrast, co‐treatment with LA/DexP‐Cy5.5 significantly reduced the intracellular green fluorescence signal, indicating that LA/DexP inhibited CpG internalization. Notably, clear yellow overlapping signals were observed within the cells, reflecting close interaction between LA/DexP nanoparticles and CpG in the intracellular environment. To quantitatively assess the cfDNA‐scavenging efficacy of LA/DexP, the concentration of residual free CpG in the cell culture supernatant was measured across treatment groups. The results indicated that the LA/DexP group showed the lowest residual CpG concentration compared to groups treated with LA or DexP alone (Figure 4b), confirming its efficient cfDNA‐scavenging capacity. To determine whether this extracellular scavenging translates into reduced intracellular DNA accumulation, we further quantified cytosolic DNA levels. As shown in Figure S24, CpG treatment increased cytosolic DNA levels compared with the control group. LA/DexP treatment significantly reduced cytosolic DNA accumulation, while LA or DexP treatment showed a modest effect. These findings suggest that LA/DexP not only scavenges extracellular cfDNA but also inhibits its cytosolic accumulation.

As cGAS is a cytosolic DNA sensor, the reduction in cytosolic DNA by LA/DexP is expected to limit the availability of cGAS ligands, thereby attenuating downstream signaling. We performed a series of experiments using CpG as a cfDNA mimetic to test this presumption. Western blot analysis showed that CpG treatment significantly upregulated the protein levels of cGAS, p‐STING and p‐TBK1, indicating activation of the cGAS‐STING pathway (Figure 4c,d). LA/DexP treatment inhibited the upregulation of all key signaling molecules and exhibited stronger inhibition than either LA or DexP alone. Correspondingly, qRT‐PCR analysis demonstrated that the mRNA levels of cGAS were downregulated after LA/DexP treatment (Figure 4e). Immunofluorescence staining showed that CpG stimulation markedly enhanced cytoplasmic fluorescence of cGAS and p‐STING, whereas co‐treatment with LA/DexP attenuated the signal intensity (Figure 4f,g). As cGAS preferentially recognizes double‐stranded DNA, ctDNA (a canonical dsDNA stimulus) was used to stimulate RAW264.7 macrophages [46]. We further validated the inhibitory effect of LA/DexP on cGAS‐STING signaling under ctDNA stimulation. Consistently, LA/DexP inhibited ctDNA‐induced cGAS‐STING pathway activation at both protein (Figure S25a) and mRNA levels (Figure S25b), compared with LA or DexP. Thus, LA/DexP inhibits cGAS‐STING pathway activation regardless of the stimulus type (CpG or ctDNA).

**FIGURE 5:**
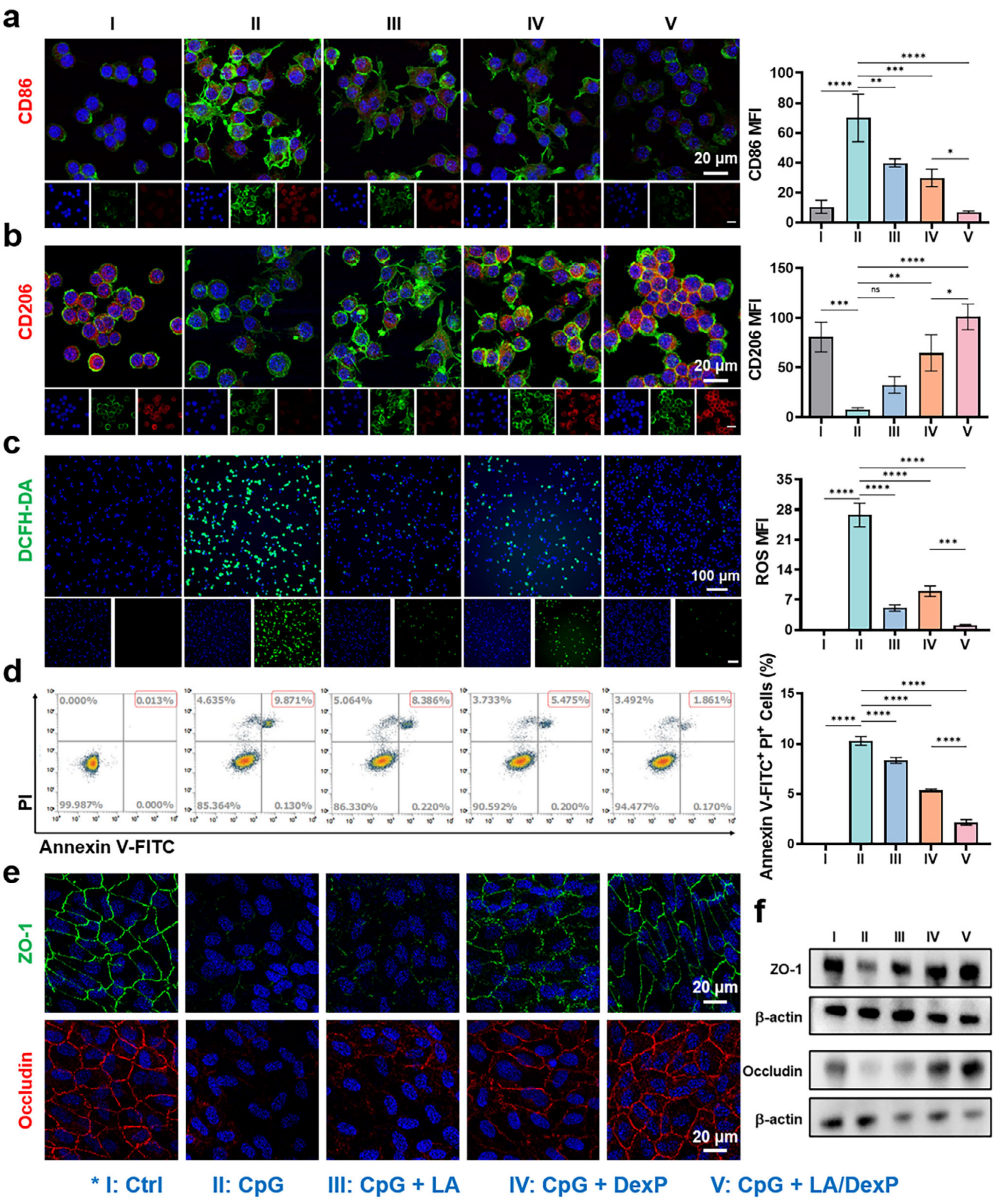
LA/DexP modulates macrophage polarization, alleviates oxidative stress, inhibits apoptosis, and protects BRB integrity in vitro. (a, b) Immunofluorescence images and MFI quantification of CD86 (a) and CD206 (b) on RAW264.7 cells under different treatments (Ctrl, CpG, CpG + LA, CpG + DexP, CpG + LA/DexP). Nuclei were stained with DAPI (blue). Scale bar: 20 µm (n = 3). (c) Representative fluorescence images and quantitative analysis of intracellular ROS levels (DCFH‐DA) in RAW264.7 cells under different treatments. Nuclei were stained with Hoechst (blue). Scale bar: 100 µm (n = 3). (d) Annexin V/PI flow cytometry plots and quantification of Annexin V^+^/PI^+^ cells in RAW264.7 cells under different treatments (n = 3). (e, f) BRB integrity assessment in ARPE‐19 cells under different treatments (PBS, CpG, CpG + LA, CpG + DexP, CpG + LA/DexP). (e) Immunofluorescence staining of ZO‐1 and occludin. Nuclei were stained with DAPI (blue). Scale bar: 20 µm. (f) Western blot analysis of ZO‐1 and occludin expression. Data are presented as mean ± SD; *p < 0.05; **p < 0.01; ***p < 0.001; ****p < 0.0001.

Activation of the cGAS‐STING pathway drives inflammatory responses and influences the balance of macrophage polarization. An imbalance characterized by excessive M1 and insufficient M2 polarization promotes the persistent progression of uveitis [47]. Restoring the M1/M2 macrophage balance has been shown to effectively alleviate uveitis [48]. We therefore examined whether LA/DexP modulates macrophage polarization in response to both CpG and ctDNA stimulation. We first assessed the expression of key factors associated with macrophage function. Western blot and qRT‐PCR analyses showed that CpG stimulation led to increased levels of pro‐inflammatory mediators (COX‐2, iNOS, TNF‐α) and decreased levels of anti‐inflammatory factors (CD206, Arg‐1) (Figure 4h,i; Figure S26). Conversely, LA/DexP treatment suppressed these pro‐inflammatory mediators and elevated the anti‐inflammatory factors, indicating its regulatory effect on macrophage polarization. To further determine the phenotypic changes, we quantified M1 (CD86^+^) and M2 (CD206^+^) macrophages by flow cytometry. CpG stimulation increased the proportion of M1 macrophages from 1.29% to 26.56% and decreased the proportion of M2 macrophages from 25.06% to 6.43%. In contrast, LA/DexP treatment reduced the M1 population to 4.33% and increased the M2 population to 32.63% (Figure S27), confirming that LA/DexP promotes macrophage repolarization from the M1 to M2 phenotype. Immunofluorescence staining further validated these findings, showing reduced CD86 intensity (Figure 5a) and enhanced CD206 intensity after LA/DexP treatment (Figure 5b). Similar to CpG treatment, ctDNA stimulation induced inflammatory responses in macrophages. ctDNA upregulated the mRNA expression of pro‐inflammatory COX‐2, iNOS and TNF‐α, and downregulated anti‐inflammatory Arg‐1 and CD206 (Figure S28a). Meanwhile, ctDNA increased the secretion of pro‐inflammatory cytokines and reduced the levels of anti‐inflammatory IL‐10 and TGF‐β (Figure S28b), accompanied by elevated CD86 staining and reduced CD206 signals in macrophages (Figure S28c). Importantly, LA/DexP treatment effectively alleviated ctDNA‐induced inflammatory imbalance, reduced CD86 signals and increased CD206 signals, thereby facilitating macrophage polarization toward the M2 phenotype.

These results demonstrate that LA/DexP not only inhibits cfDNA‐induced activation of the cGAS‐STING pathway, but also promotes macrophage polarization from a pro‐inflammatory M1 phenotype to an anti‐inflammatory M2 phenotype, highlighting its role in ameliorating the inflammatory microenvironment in uveitis.

### 2.6 LA/DexP Attenuates cfDNA‐Induced Oxidative Stress, Inhibits cfDNA‐Triggered Apoptosis, and Protects BRB Integrity In Vitro

Oxidative stress is widely recognized as a key risk factor in exacerbating retinal inflammation in uveitis [49]. While ROS can promote cfDNA release, whether cfDNA itself further amplifies oxidative stress has not yet been determined. To evaluate the effect of LA/DexP on intracellular oxidative stress, RAW264.7 cells were stimulated with CpG and treated with LA, DexP, or LA/DexP for 12 h. Intracellular ROS levels were assessed using the 2',7'‐dichlorodihydrofluorescein diacetate (DCFH‐DA) probe. CpG stimulation markedly increased green fluorescence, indicating successful induction of oxidative stress (Figure 5c). Treatment with LA/DexP significantly attenuated this fluorescence signal, exhibiting superior efficacy compared to LA or DexP alone, which is ascribed to its potent ROS‐scavenging capacity. This scavenging effect was further quantified by flow cytometry, which verified that LA/DexP most effectively reduced intracellular ROS levels (Figure S29a). Intracellular malondialdehyde (MDA), a classic marker of lipid peroxidation [50], and superoxide dismutase (SOD), a key endogenous antioxidant enzyme [51], were subsequently analyzed. The results revealed that CpG stimulation significantly increased MDA content and decreased SOD activity, whereas LA/DexP treatment reduced MDA levels and restored SOD activity (Figure S29b). These results indicate that LA/DexP alleviates cfDNA‐induced intracellular oxidative stress injury, as evidenced by reduced ROS levels, decreased lipid peroxidation, and restored SOD activity.

Abnormal apoptosis leads to structural damage of the retina and choroid [52]. Inhibiting pathological apoptosis is essential for reducing disease‐related injury. For this purpose, we examined the anti‐apoptotic efficacy of LA/DexP. Annexin V/PI flow cytometry analysis showed that CpG stimulation increased the apoptotic rate from 0.01% in the control group to 9.87%. Treatment with LA/DexP reduced the apoptotic rate to 1.86%, which was significantly lower than that in the LA (8.39%) or DexP (5.47%) groups (Figure 5d). Correspondingly, TUNEL staining showed that compared with other treatment groups, the positive signal in the LA/DexP group was markedly reduced, indicating that LA/DexP attenuates DNA fragmentation damage (Figure S29c). These data demonstrate that LA/DexP effectively inhibits apoptosis by suppressing cfDNA‐induced pathological signals.

BRB disruption is a core pathological feature of uveitis [5, 53]. The loss of its structural integrity directly increases vascular permeability, promotes immune cell infiltration, and sustains chronic inflammation. Restoring BRB integrity is thus a key therapeutic objective for curbing disease progression. To investigate the effect of CpG stimulation on the BRB, ARPE‐19 cells were exposed to CpG and treated with LA, DexP, or LA/DexP for 12 h. The localization and expression of tight junction proteins ZO‐1 and occludin were assessed. The results revealed that CpG treatment disrupted the normal continuous, linear distribution of ZO‐1 and occludin, leading to a diffuse and discontinuous distribution pattern (Figure 5e). This change was accompanied by a downregulation of protein levels (Figure 5f; Figure S30), suggesting that CpG treatment impaired tight‐junction integrity. In contrast, LA/DexP treatment preserved the normal linear arrangement of these proteins at cell borders and upregulated their expression, suggesting that LA/DexP protects BRB integrity by mitigating CpG‐induced tight junction protein damage.

In summary, our findings demonstrate that cfDNA can induce oxidative stress, apoptosis, and disruption of the blood‐retinal barrier, while LA/DexP effectively alleviates these pathological processes through a multi‐target synergistic mechanism.

### 2.7 LA/DexP Reduces EAU Inflammation and Pathological Progression

**FIGURE 6:**
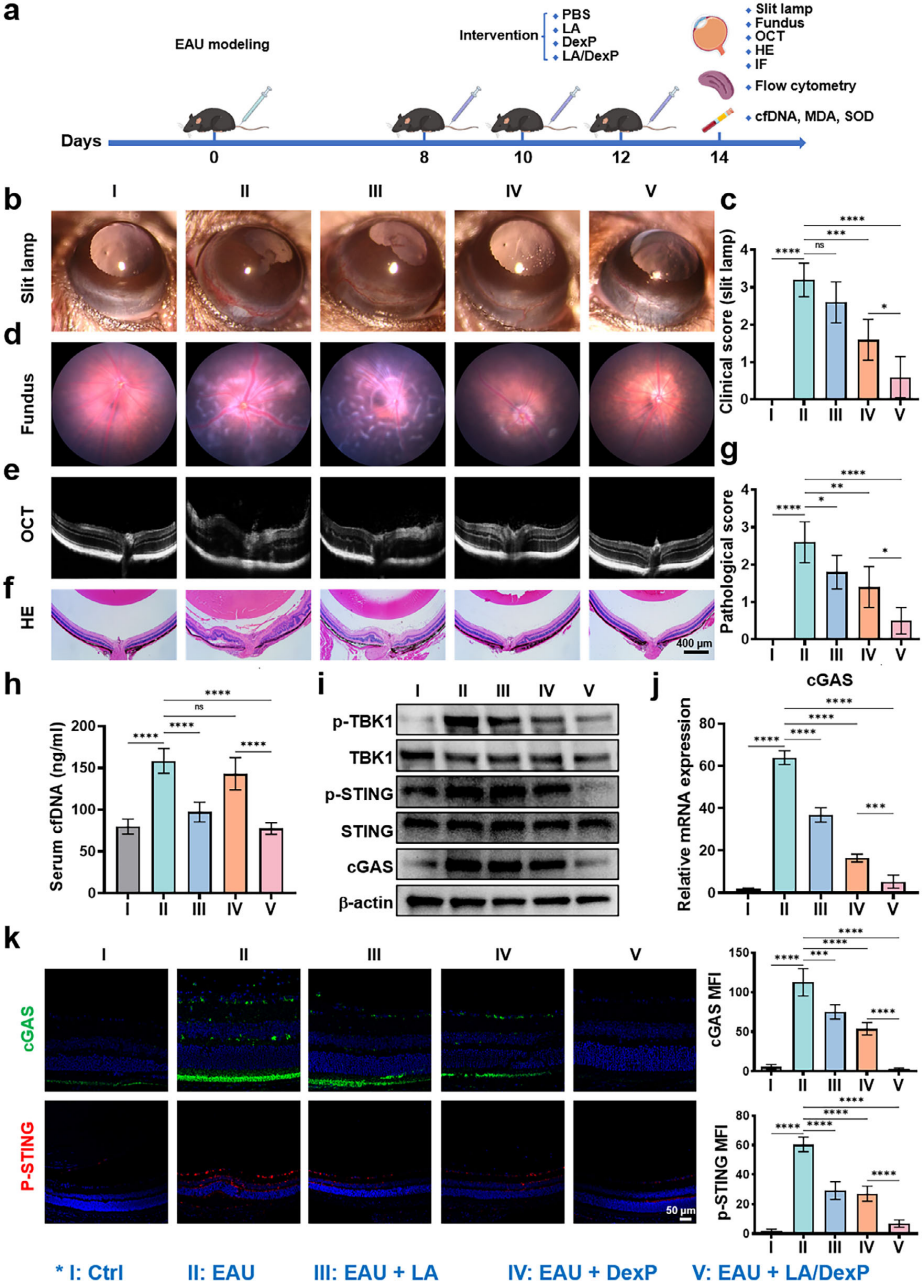
LA/DexP exhibits potent therapeutic efficacy and inhibits the retinal cGAS‐STING pathway in EAU. (a) Schematic illustration of EAU induction, treatment regimen, and evaluation timeline. (b) Representative slit‐lamp biomicroscopy images of each group (Ctrl, EAU, EAU+LA, EAU+DexP, EAU+LA/DexP) on day 14 post‐immunization. (c) Clinical scores of inflammation based on slit‐lamp biomicroscopy images (n = 5). (d) Representative fundus photography images. (e) Representative OCT images. (f) Representative H&E‐stained retinal sections. (g) Histopathological scores based on retinal H&E sections (n = 5). (h) Serum cfDNA levels in different treatment groups (n = 5). (i) Representative Western blot images of cGAS, p‐STING, and p‐TBK1 protein levels in retinal tissues. (j) qRT‐PCR analysis of cGAS expression in retinal tissues (n = 4). (k) Immunofluorescence staining and quantification of cGAS and STING in retinal cryosections (n = 4). Nuclei were stained with DAPI (blue). Scale bar: 50 µm. Data are presented as mean ± SD; *p < 0.05; **p < 0.01; ***p < 0.001; ****p < 0.0001.

It is well known that systemic glucocorticoids and other immunosuppressive agents are widely used in patients with intermediate, posterior or generalized uveitis based on disease pathogenesis [54]. In AU, beyond conventional systemic inflammatory responses, we observed increased circulating cfDNA in patient serum. Local ocular treatment alone cannot clear these circulating molecules, so we tested the therapeutic effect of intravenously injected LA/DexP in the EAU mouse model. Specifically, on day 8 post‐immunization, mice were treated intravenously with PBS, LA, DexP, or LA/DexP. On day 14, ocular inflammation was assessed by slit‐lamp examination, fundus photography, and optical coherence tomography (OCT). Subsequently, retinal tissues, spleen, and serum were collected for histopathological, molecular, and biochemical analyses (Figure 6a). Slit‐lamp examination revealed severe inflammation in the EAU group, characterized by marked ciliary and conjunctival hyperemia and posterior iris synechiae (Figure 6b), accompanied by significantly elevated clinical scores (Figure 6c). Fundus imaging showed tortuous retinal vessels with gray‐white exudates (Figure 6d). In contrast, LA/DexP treatment effectively mitigated intraocular inflammation, as evidenced by mild conjunctival congestion, absence of synechiae, normal retinal vessels, and significantly reduced clinical scores.

To further examine the retinal microstructure, OCT analysis was performed. The results showed that the retina of EAU mice displayed blurred layers and focal areas of architectural distortion, suggesting the presence of inflammatory edema and structural damage (Figure 6e). After LA/DexP treatment, the retinal layering became clear, and normal retinal architecture was restored. To confirm these findings at the histological level, hematoxylin and eosin (H&E) staining was performed. This analysis revealed severe structural disorder and a large number of infiltrating inflammatory cells in the retina of EAU mice (Figure 6f), and the histopathological score was higher compared to that of normal mice (Figure 6g). After LA/DexP treatment, retinal structure was restored, with clear layers and absence of inflammatory infiltration, resulting in a pathological score significantly lower than that of the EAU group. Although treatment with LA or DexP alone achieved partial alleviation of inflammation, they failed to fully restore the retinal structure. Overall, compared to the other treatment groups, LA/DexP demonstrated superior in vivo efficacy in suppressing intraocular inflammation and promoting retinal structural repair in EAU.

### 2.8 LA/DexP Mitigates EAU by Scavenging cfDNA and Inhibiting the cGAS‐STING Pathway Activation

To evaluate whether LA/DexP modulates the cGAS‐STING pathway in vivo, we first measured serum levels of cfDNA in EAU mice. As shown in Figure 6h, serum cfDNA levels were significantly elevated in EAU mice compared with normal controls. LA/DexP treatment effectively reduced serum cfDNA to near‐normal levels, confirming its cfDNA‐scavenging efficacy under disease conditions. This systemic clearance should limit cfDNA entry into the retina. To test this, we next assessed the activation status of the cGAS‐STING signaling axis in retinal tissues by Western blot. Protein expression of cGAS, p‐STING and p‐TBK1 was markedly upregulated in EAU retinas compared with controls (Figure 6i; Figure S31), indicating that the cGAS‐STING pathway was activated during uveitis. Treatment with LA/DexP downregulated the expression of these proteins, effectively reversing this abnormal activation. Notably, LA/DexP exerted a stronger inhibitory effect than either LA or DexP alone. Further evidence came from qRT‐PCR analysis showing increased mRNA levels of cGAS in EAU retinas, with a significant reduction observed after LA/DexP treatment (Figure 6j). Consistent with these data, immunofluorescence staining revealed that LA/DexP treatment led to the lowest expression of cGAS and p‐STING relative to the other groups (Figure 6k). Collectively, these data demonstrate that LA/DexP inhibits the aberrant activation of the cGAS‐STING pathway in the retina through scavenging pathological cfDNA, thereby contributing to its therapeutic efficacy in EAU.

### 2.9 LA/DexP Reshapes the Immune Microenvironment in Vivo via Modulation of Macrophage Polarization and Th1/Th17‐Treg Balance

**FIGURE 7:**
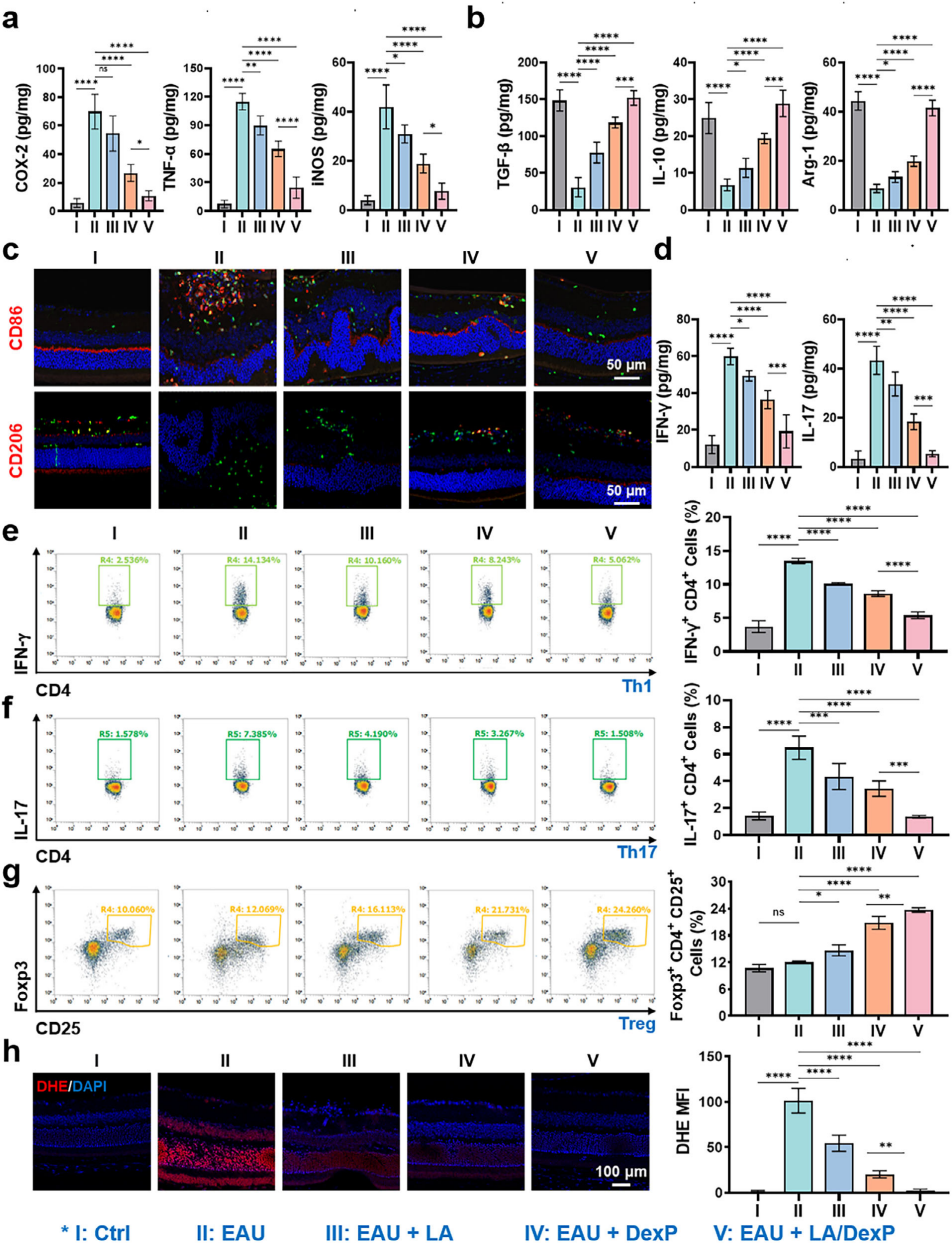
LA/DexP modulates the immune microenvironment and mitigates oxidative stress in EAU. (a) Retinal protein levels of COX‐2, iNOS, and TNF‐α measured by ELISA in different treatment groups (Ctrl, EAU, EAU+LA, EAU+DexP, EAU+LA/DexP) (n = 5). (b) Retinal protein levels of TGF‐β, IL‐10, and Arg‐1 measured by ELISA (n = 5). (c) Representative immunofluorescence images of F4/80 co‐localized with CD86 or CD206 in retinal cryosections. Nuclei were stained with DAPI (blue). Scale bar: 50 µm. (d) Retinal protein levels of IFN‐γ and IL‐17 measured by ELISA (n = 5). (e‐g) Flow cytometric analysis of splenic CD4^+^ T‐cell subsets: (e) Th1 (CD4^+^IFN‐γ^+^) (n = 5), (f) Th17 (CD4^+^IL‐17^+^) (n = 5), and (g) Treg (CD4^+^CD25^+^Foxp3^+^) (n = 3). (h) DHE staining and quantification of retinal ROS levels (n = 4). Nuclei were stained with DAPI (blue). Scale bar: 100 µm. Data are presented as mean ± SD; *p < 0.05; **p < 0.01; ***p < 0.001; ****p < 0.0001.

Macrophages, as core effector cells of innate immunity, play a critical role in maintaining immune homeostasis in uveitis through their polarization balance. To evaluate the immunomodulatory effect of LA/DexP on macrophages in vivo, we measured the levels of key macrophage‐related cytokines in retinal tissues and serum by enzyme‐linked immunosorbent assay (ELISA). The results showed that pro‐inflammatory factors (TNF‐α, COX‐2, iNOS; Figure 7a; Figure S32a) were increased, while anti‐inflammatory cytokines (TGF‐β, IL‐10, Arg‐1; Figure 7b; Figure S32b) were decreased in both ocular tissues and serum of EAU mice, indicating a tendency toward M1 polarization during EAU progression. LA/DexP treatment effectively suppressed the expression of pro‐inflammatory factors and restored anti‐inflammatory cytokine levels, suggesting its potential to promote macrophage polarization toward the M2 phenotype. Next, we performed co‐localization immunofluorescence staining for macrophage marker F4/80 with M1 marker CD86 and M2 marker CD206 in retinal tissues. Compared with normal controls, the EAU group displayed more F4/80^+^CD86^+^ cells and fewer F4/80^+^CD206^+^ cells, indicating a shift toward the pro‐inflammatory macrophage phenotype (Figure 7c). Conversely, LA/DexP treatment reduced F4/80^+^CD86^+^ cells and increased F4/80^+^CD206^+^ cells, suggesting a transition toward the M2 phenotype. Collectively, these data indicate that LA/DexP promotes macrophage polarization toward the anti‐inflammatory M2 phenotype in vivo.

The imbalance between pathogenic T cell subsets, including T helper 1 (Th1) and T helper 17 (Th17) cells, and immunosuppressive regulatory T (Treg) cells is another key factor that disrupts immune tolerance in uveitis [55]. IFN‐γ and IL‐17 are major effector cytokines secreted by Th1 and Th17 cells, respectively [56]. To determine whether LA/DexP modulates adaptive immunity, we first assessed its effect on the expression of these pro‐inflammatory cytokines. ELISA results revealed that IFN‐γ and IL‐17A levels were significantly elevated in both retinal tissues and serum of EAU mice compared with normal controls (Figure 7d; Figure S33). LA/DexP treatment exhibited superior inhibitory effects on these cytokines compared to LA or DexP alone (Figure 7d), suggesting that LA/DexP may exert immunomodulatory effects by regulating T cell differentiation. To directly test this hypothesis, we analyzed splenic T cell subsets by flow cytometry. Compared with normal controls (2.54%), EAU mice displayed a marked expansion of Th1 cells (14.13%), which was significantly reduced by LA/DexP treatment (5.06%) (Figure 7e). Similarly, the proportion of Th17 cells increased from 1.58% in normal controls to 7.38% in EAU mice, and was reduced to 1.51% after LA/DexP treatment (Figure 7f). Notably, the Treg proportion, which remained unchanged in EAU mice, was markedly elevated to 24.26% following LA/DexP treatment (Figure 7g). These results demonstrate that LA/DexP inhibits the expansion of pathogenic Th1/Th17 cells while promoting the induction and differentiation of Treg cells.

Taken together, our findings reveal that LA/DexP reshapes the immune microenvironment in vivo through a dual mechanism. On one hand, it drives macrophage repolarization from the M1 to M2 phenotype, modulating local innate immune responses. On the other hand, it restores the Th1/Th17‐Treg balance to reestablish systemic immune homeostasis.

### 2.10 LA/DexP Attenuates Retinal Damage in EAU by Attenuating Oxidative Stress, Inhibiting Apoptosis, and Preserving Barrier Integrity

**FIGURE 8:**
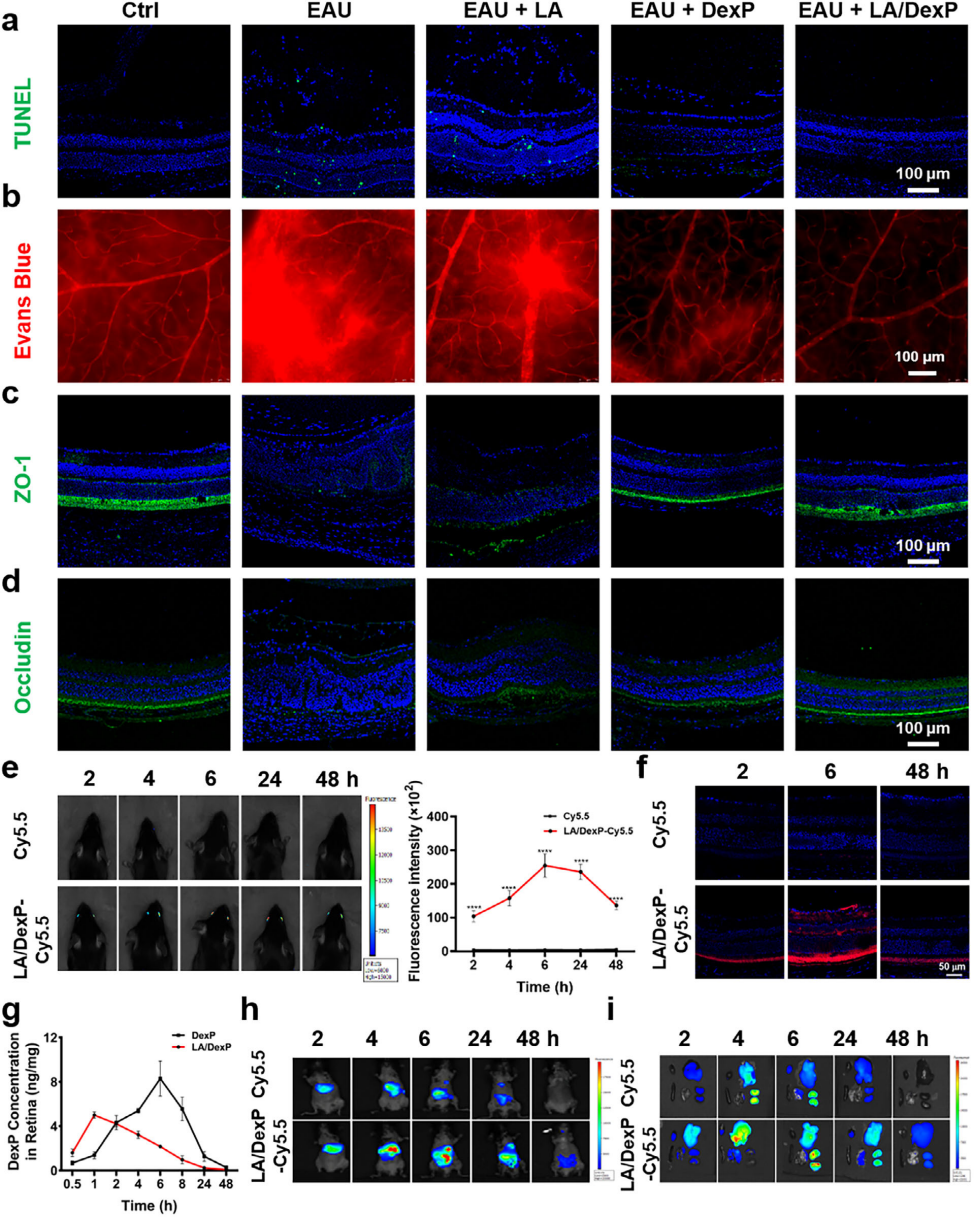
LA/DexP suppresses apoptosis, preserves the blood‐retinal barrier, and exhibits efficient delivery and biocompatibility in vivo. (a) TUNEL staining images of retinal sections from different treatment groups (Ctrl, EAU, EAU+LA, EAU+DexP, EAU+LA/DexP). Nuclei were stained with DAPI (blue). Scale bar: 100 µm. (b) Representative Evans Blue leakage images of retinal vessels. (c, d) Representative immunofluorescence images of ZO‐1 (c) and occludin (d) in retinal sections. Nuclei were stained with DAPI (blue). Scale bar: 100 µm. (e) In vivo fluorescence imaging of the eye region at 2, 4, 6, 24, and 48 h after intravenous injection of free Cy5.5 or LA/DexP‐Cy5.5. (f) Confocal fluorescence images of retinal sections at 2, 6, and 48 h after intravenous injection of free Cy5.5 or LA/DexP‐Cy5.5. Nuclei were stained with DAPI (blue). Scale bar: 50 µm. (g) LC‐MS/MS quantification of DexP concentrations in retinal tissues at different time points following intravenous administration of free DexP or LA/DexP (n = 3). (h) Whole‐body in vivo fluorescence images acquired at 2, 4, 6, 24, and 48 h post intravenous injection. (i) *Ex vivo* fluorescence imaging of major organs (heart, liver, spleen, lung, and kidneys) at different time points. Data are presented as mean ± SD. ****p < 0.0001.

Previous in vitro studies have confirmed that LA/DexP possesses multiple functions, including ROS scavenging, apoptosis inhibition, and tight junction protein protection. Based on these findings, we further evaluated its retinal protective effects in an EAU mouse model. Dihydroethidium (DHE) fluorescence staining revealed weak ROS signals in the retinas of normal mice (Figure 7h), whereas intense red fluorescence was observed across all retinal layers in EAU mice, indicating elevated oxidative stress under disease conditions. LA/DexP treatment markedly reduced retinal ROS fluorescence, showing superior efficacy compared to LA or DexP monotherapy. Consistently, LA/DexP treatment effectively reversed the increase in MDA content and the decrease in SOD activity observed in EAU mouse retinas (Figure S34). These results demonstrate that LA/DexP alleviates retinal oxidative stress injury in the EAU model. TUNEL staining showed abundant positive signals in the retinas of EAU mice, while few were detected in normal controls. LA/DexP treatment reduced these positive signals (Figure 8a), confirming its inhibitory effect on pathological apoptosis in vivo.

We next examined the protective effect of LA/DexP on BRB integrity using Evans blue perfusion and ZO‐1/occludin immunofluorescence staining. Evans blue retinal imaging revealed obvious dye leakage in retinal vessels of EAU mice, indicating severe impairment of the BRB. Among all treatment groups, the LA/DexP group showed the mildest dye leakage (Figure 8b). Quantitative measurement of Evans blue extravasation further confirmed that LA/DexP alleviated retinal leakage more effectively than LA or DexP (Figure S35). Immunofluorescence analysis showed that ZO‐1 and occludin exhibited a continuous linear distribution in the normal group, whereas their distribution became diffuse and discontinuous in the EAU group (Figure 8c,d). LA/DexP treatment restored the continuous distribution of both proteins, further confirming its protective effect in reducing BRB disruption. Therefore, LA/DexP exerts its retinal protective effects at multiple levels, including alleviating oxidative stress, inhibiting apoptosis, and preserving BRB integrity.

### 2.11 LA/DexP Demonstrates BRB‐Penetrating Capability and Good In‐Vivo Biocompatibility

Favorable penetration of the BRB and good in vivo biocompatibility are prerequisites for LA/DexP to exert its potent anti‐inflammatory and retinal protective effects. To evaluate its delivery performance, we compared the spatiotemporal distribution of LA/DexP‐Cy5.5 with that of free Cy5.5 in healthy mice. In vivo fluorescence imaging revealed detectable ocular signals within 2 h after intravenous injection of LA/DexP‐Cy5.5, which peaked at 6 h, remained at a high level at 24 h, and were still detectable at 48 h (Figure 8e). This ocular accumulation was related to its amphiphilic structure and thiol‐mediated barrier penetration. In contrast, free Cy5.5, due to its high hydrophilicity and poor membrane affinity, was unable to cross the BRB via passive diffusion and showed negligible ocular accumulation. *Ex vivo* retinal fluorescence imaging further confirmed significant enrichment of LA/DexP‐Cy5.5 in retinal tissue (Figure S36). Confocal microscopy of retinal sections demonstrated that LA/DexP‐Cy5.5 was present in the retina at 2 h, showed strong fluorescence across all retinal layers at 6 h, and retained detectable signals at 48 h (Figure 8f).

To further quantify the retinal enrichment capacity of LA/DexP, we measured retinal DexP concentrations after intravenous administration via liquid chromatography‐tandem mass spectrometry (LC‐MS/MS) and generated concentration–time curves (Figure 8g). Pharmacokinetic parameters were calculated using non‐compartmental analysis (Table S2). Free DexP reached its peak concentration rapidly (T_max_ = 1 h, C_max_ = 4.99 ± 0.27 ng/mg), followed by fast elimination (t_1/2_ = 2.81 h). In contrast, LA/DexP markedly altered the retinal distribution and elimination kinetics of DexP, with a delayed T_max_ of 6 h, a 1.66‐fold higher C_max_ than free DexP, and a 3.84‐fold increase in retinal drug levels at 6 h relative to free DexP. Non‐compartmental calculations further showed that LA/DexP exhibited prolonged pharmacokinetic profiles in retinal tissue. Its elimination half‐life (11.09 h), AUC_0‐∞_ (119.76 ng·h/mg) and MRT_0‐∞_ (15.90 h) were 3.95‐, 3.03‐ and 3.88‐fold greater than those of free DexP, respectively. This extended half‐life and improved retinal accumulation support sustained intraocular drug levels, suggesting that the LA/DexP nanoplatform improves the retinal bioavailability and intraocular retention of DexP. Furthermore, whole‐body and *ex vivo* organ fluorescence imaging tracking intravenously administered LA/DexP‐Cy5.5 showed that the fluorescence signals were predominantly localized to the liver, with signals declining gradually after 4 h (Figure 8h,i; Figure S37), reflecting clearance via the mononuclear phagocyte system. Compared with free Cy5.5, LA/DexP‐Cy5.5 exhibited stronger hepatic fluorescence and slower clearance, suggesting an advantage in prolonged circulation.

In parallel, systemic biocompatibility assessments were conducted. The hemolysis assay revealed that LA/DexP at concentrations ranging from 0 to 500 µg/mL did not induce hemolysis of red blood cells (Figure S38). Even at the highest concentration tested, the hemolysis rate remained below the safety threshold of 5%. At 14 days after intravenous administration of LA, DexP or LA/DexP, all measured parameters, including complete blood counts, liver function markers (ALT, AST, T‐BIL), and renal function markers (BUN, CREA, UA), remained within normal physiological ranges (Figure S39). Furthermore, H&E staining of major organs (heart, liver, spleen, lungs, kidneys) showed no evidence of inflammatory infiltration, necrosis, or other pathological changes (Figure S40). Collectively, these findings demonstrate that LA/DexP achieves efficient ocular accumulation while maintaining excellent systemic biocompatibility, highlighting its potential as a safe and effective nano‐therapeutic platform.

## 3 Conclusion

This study developed a cationic poly(disulfide)‐drug nanoplatform (LA/DexP) for the synergistic treatment of autoimmune uveitis. The platform was shown to overcome the limitations of conventional delivery strategies by penetrating the blood‐retinal barrier and enhancing the ocular bioavailability of DexP. Furthermore, it effectively scavenged cfDNA and inhibited the overactivated cGAS‐STING inflammatory cascade. In the EAU model, LA/DexP treatment effectively alleviated clinical symptoms and pathological damage, while improving the immune microenvironment. The platform also exhibited favorable biocompatibility, with no observable systemic toxicity. In conclusion, this work demonstrates the feasibility of a “drug delivery‐pathway inhibition” synergistic strategy, offering a new perspective for the treatment of AU and other autoimmune diseases mediated by aberrant nucleic acid‐sensing pathways.

## 4 Experimental Section

### 4.1 Materials

α‐Lipoic acid, 1,1'‐Carbonyldiimidazole (CDI), L‐Arginine methyl ester dihydrochloride, N,N‐Dimethylformamide (DMF), N,N‐Diisopropylethylamine (DIEA), triethylamine, and isopropyl ether were purchased from Macklin Biochemical Co., Ltd. (Shanghai, China). DTNB and DexP were obtained from Aladdin Biochemical Technology Co., Ltd. (Shanghai, China). Cyanine 5.5 carboxylic acid and SYBR Green Master Mix were sourced from MedChemExpress (Monmouth Junction, NJ, USA). The SteadyPure Universal RNA Extraction Kit and Evo M‐MLV RT Kit with gDNA Clean were purchased from Precision Biotechnology Co., Ltd. (Changsha, China). ctDNA was purchased from Solarbio Science & Technology Co., Ltd. (Beijing, China). The dsDNA HS Assay Kit was acquired from Yeasen Biotechnology Co., Ltd. (Shanghai, China). The human retinal pigment epithelial cell line (ARPE‐19, RRID:CVCL_0145), murine macrophage cell line (RAW264.7, RRID:CVCL_0493), and murine fibroblast cell line (L929, RRID: CVCL_0462) were purchased from Procell Life Technology Co., Ltd. (Wuhan, China). Complete Freund's adjuvant (CFA) was provided by Sigma‐Aldrich (St. Louis, MO, USA). The IRBP peptide (human, residues 650–670, sequence LAQGAYRTAVDLESLASQLT) and the CpG oligodeoxynucleotide 1826 (CpG, sequence TCC ATG ACG TTC CTG ACG TT) were synthesized by Sangon Biotech Co., Ltd. (Shanghai, China). The ELISA kits were obtained from Mengbio Biotechnology Co., Ltd. (Chongqing, China). The Cell‐free DNA Isolation Kit with Magnetic Beads, CCK‐8, Total Superoxide Dismutase Activity Assay Kit, Lipid Peroxidation Assay Kit, Tris‐HCl buffer (pH 7.5), DCFH‐DA and other reagents were purchased from Beyotime Biotechnology Co., Ltd. (Shanghai, China).

### 4.2 Patient Samples

Serum samples used in this study were obtained from the Biobank of the First Affiliated Hospital of Chongqing Medical University, including 30 patients with active uveitis and 10 age‐ and sex‐matched healthy controls. Among the patients, 20 were diagnosed with BD and 10 with VKH disease. The patient cohort consisted of 18 males and 12 females, with ages ranging from 18 to 62 years. All patients met the corresponding international criteria for diagnostic of BD [57] or VKH [58] and had not taken any immunosuppressive drugs for at least one week before blood collection. Written informed consent was obtained from every participant. This study was performed in accordance with the Declaration of Helsinki and received approval from the Ethics Research Committee of the First Affiliated Hospital of Chongqing Medical University (approval no. ZZ2024‐569‐02).

### 4.3 Animals

Mice (Female, C57BL/6J, 6–8 weeks) were sourced from Enbi Biotechnology Co., Ltd. (Chongqing, China) and maintained in a specific pathogen‐free facility. All experimental protocols received approval from the Animal Ethics Committee of the First Affiliated Hospital of Chongqing Medical University (approval no. 2021–222) and adhered to the ARVO Statement on the Use of Animals in Ophthalmology and Vision Research.

### 4.4 Establishment and Evaluation of the EAU Model

Mice were immunized to induce EAU by subcutaneous injection at multiple sites (bilateral axillae, inguinal regions, back, neck, and tail base) with an emulsion containing human IRBP651‐670 peptide (500 µg/mL) and CFA (5 mg/mL Mycobacterium tuberculosis H37Ra). Each mouse also received an intraperitoneal injection of pertussis toxin (1 µg). Successful induction was confirmed on day 7 by slit‐lamp observation of intraocular inflammation. Immunized mice were then randomly assigned to the following groups: EAU, EAU + LA, EAU + DexP, and EAU + LA/DexP, with unimmunized mice serving as healthy controls. From day 8 post‐immunization, mice received tail‐vein injections of the corresponding drug (2 mg/kg) or PBS every 48 h. The doses of DexP were normalized to the amount contained in LA/DexP based on its drug loading content. Ocular inflammation was evaluated on day 14 using slit‐lamp biomicroscopy, fundus imaging, and OCT. Clinical and histopathological scoring was performed in a blinded manner according to established criteria [59, 60]. Evans blue perfusion was performed to evaluate BRB integrity, assessed by both retinal imaging and absorbance quantification at 620 nm. Following assessment, serum was collected for measurement of circulating cfDNA, MDA levels, and SOD activity. Eyeballs were enucleated for further analysis, including H&E staining, DHE staining, and immunofluorescence staining for related cytokines. For RNA‐Seq analysis, retinal tissues from normal and EAU mice were collected for total RNA extraction. Sequencing was performed by Lianchuan Biotechnology Co., Ltd., and all data were analyzed through its online platform (https://www.omicstudio.cn/tool).

### 4.5 CfDNA Extraction and Measurement

Blood samples from EAU mice were clotted for 1 h at room temperature. Serum was separated by centrifugation at 2,000 ×g (4°C, 15 min), cleared by a second centrifugation at 12,000 ×g (4°C, 10 min), aliquoted, and stored at −80°C. Human serum was processed similarly. cfDNA was isolated from 100 µL serum using a Cell‐free DNA Isolation Kit with Magnetic Beads, and its concentration was determined with a high‐sensitivity dsDNA HS Assay Kit.

### 4.6 Synthesis and Characterization of La

The synthesis of LA followed a reported method [32]. Briefly, α‐Lipoic acid (412 mg, 2 mmol) was dissolved in anhydrous DMF (4 mL) and CDI (324 mg, 2 mmol) was added. The mixture was stirred under nitrogen at room temperature for 2 h to activate the carboxyl group. Subsequently, a solution of L‐Aarginine methyl ester dihydrochloride (261 mg) and DIEA (174 µL) in anhydrous DMF (4 mL) was prepared, filtered through cotton, and added to the reaction mixture. Stirring was continued under the same conditions for 3.5 h. Upon completion, the product was precipitated by dropwise addition of the reaction mixture into isopropyl ether (20 mL). The resulting solid was collected by centrifugation, washed three times with a DCM/isopropyl ether mixture (1:2, v/v, 20 mL each), and dried under vacuum to obtain the guanidyl‐functionalized monomer. The structure and molecular weight of LA were analyzed by ^1^H NMR spectroscopy (Bruker AV, 400 MHz; DMSO‐d_6_) and MALDI‐TOF MS (Bruker ultraflextreme), respectively.

### 4.7 Synthesis and Characterization of LA/DexP

LA/DexP nanoparticles were prepared by mixing an aqueous solution of the disulfide‐containing monomer LA (10 mg/mL, 20 mM) with an equal volume (4.0 mL) of DexP in 20 mM Tris‐HCl buffer (pH 7.5) at 37°C. The mixture was stirred at room temperature for 6 h. The resulting product was then purified via dialysis against water for 24 h to remove unreacted monomers and free DexP, followed by lyophilization for 48 h to obtain the nanoparticles. To optimize the assembly, formulations with varying LA:DexP molar ratios (10:1, 25:1, and 50:1) were prepared accordingly.

The chemical structure of the monomer was confirmed by ^1^H NMR spectroscopy using a Bruker Avance III HD spectrometer. FT‐IR spectra were recorded on a Thermo Scientific Nicolet iS5 spectrometer, and UV–vis absorption spectra were obtained using an Implen NanoPhotometer N50 spectrophotometer. The nanoparticle morphology was observed using a JEOL JEM F200 TEM. The particle size distribution and zeta potential were measured with a Malvern Zetasizer Nano ZS90 laser particle size analyzer based on DLS. Furthermore, the stability was assessed by monitoring changes in particle size after dispersion in different media, including water, PBS, and DMEM supplemented with 10% FBS, over time points of 0, 3, 7, 14, and 21 days.

### 4.8 Molecular Dynamics Simulations

Molecular dynamics simulations were performed using Materials Studio software to investigate the co‐assembly behavior of cationic and anionic species driven by electrostatic interactions and the dynamic evolution of disulfide bonds. All simulations were conducted under the NPT ensemble using the COMPASS III force field, with the temperature and pressure maintained at 298 K and 1 bar, respectively. A periodic simulation box of approximately 41 Å × 41 Å × 41 Å was constructed according to the target stoichiometry. After energy minimization, a 500‐ps relaxation molecular dynamics simulation was performed with a time step of 1 fs. Temperature and pressure were controlled using the Nosé–Hoover thermostat and the Berendsen barostat, respectively. The cutoff distance for non‐bonded interactions was set to 12.5 Å. The formation of S–S bonds was monitored during the simulation. For quantitative analysis, 11 trajectory frames spanning from 1 to 500 ps were extracted from the relaxation trajectory. Hydrogen‐bond counts were obtained using a custom script after removing water molecules, and the solvent‐accessible surface area was calculated with a probe radius of 1.4 Å. A detailed local snapshot was taken from the final conFiguration at 500 ps, while the structural evolution was illustrated using six representative instantaneous conFigurations at 1, 100, 200, 300, 400, and 500 ps.

### 4.9 Agarose Gel Electrophoresis

DNA models, including CpG, ctDNA, and a DNA ladder (200–12,000 bp), were incubated with LA/DexP (0–500 µg/mL) for 2 h at room temperature. Samples (10 µL) were then electrophoresed on agarose gels (100 V, 30 min) in 1× TAE buffer and imaged using an Amersham Imager 6000 system.

### 4.10 Ethidium Bromide Competitive Binding Assay

The DNA‐EtBr complex was formed by mixing CpG and EtBr solutions (100 µg/mL each, 4 µL). Different concentrations of LA/DexP were added and adjusted to 160 µL with PBS, followed by incubation at 37°C for 24 h. Fluorescence intensity was measured at 520 nm excitation, and the binding efficiency was calculated as BE% = [1 – (A A_0_)/(A_1_ – A_0_)] × 100%, where A, A_0_, and A_1_ denote the fluorescence of the sample, free EtBr, and the initial complex, respectively. An experiment performed in 10% FBS diluted in PBS was conducted in parallel to evaluate anti‐protein interference.

### 4.11 In Vitro Antioxidant Activity Assay

The in vitro antioxidant activity of LA/DexP was evaluated using commercial assay kits according to the manufacturers’ protocols. The scavenging capacities against DPPH•, ABTS•^+^, O_2_•^−^, and •OH were measured. The scavenging percentage was calculated using the formula: Scavenging Activity (%) = [(Acontrol Asample) / Acontrol] × 100%, where Acontrol and Asample represent the absorbance of the control (without LA/DexP) and the sample, respectively. Additionally, a hydrogen peroxide (H_2_O_2_) assay kit was employed to evaluate the effect of LA/DexP on H_2_O_2_ levels in vitro, by measuring the changes in fluorescence intensity as a function of time and LA/DexP concentration to monitor the H_2_O_2_ scavenging behavior.

### 4.12 In Vitro Drug Release Study

The encapsulation efficiency and drug loading content of LA/DexP were determined following previously reported methods [61]. For the drug release study, LA/DexP nanoparticles were loaded into dialysis bags and incubated in PBS (pH 7.4) containing 0, 0.01 mM, 0.05 mM, 0.1 mM, 0.5 mM, or 1 mM H_2_O_2_ at 37°C with shaking. At designated intervals, 1 mL of the external medium was sampled and replaced with fresh medium of equal volume and H_2_O_2_ concentration. The DexP concentration was determined by absorbance at 240 nm (Implen NanoPhotometer N50). Cumulative release was calculated as: Cumulative Release (%) = (Σ (C_n_ × V) / M_0_) × 100%, where C_n_ is the DexP concentration at time “n”, V is the total medium volume, and M_0_ is the total DexP loaded. Additionally, after 12 h of incubation with 1 mM H_2_O_2_, the particle size and zeta potential of LA/DexP were measured by DLS, and morphological changes were observed by TEM.

### 4.13 Cell Culture and Cytotoxicity Testing

The RAW264.7, ARPE‐19, and L929 cell lines were cultured in their respective complete media under standard conditions (37°C, 5% CO_2_, 95% humidity). Cells in the logarithmic growth phase were seeded into 96‐well plates at a density of 1 × 10^5^ cells per well. Following adherence, cells were treated with 100 µL of fresh medium containing different concentrations of LA or LA/DexP and incubated for 24 h or 48 h. Cell viability and cytotoxicity were respectively assessed using the CCK‐8 assay and Calcein‐AM/PI double‐staining kit.

### 4.14 In Vitro BRB Penetration

An in vitro model of the BRB was established using a confluent monolayer of ARPE‐19 cells. The cells were seeded onto polyester Transwell inserts (6 mm diameter, 0.33 cm^2^ membrane area, 0.4 µm pore size; Corning, USA) at a density of 1×10^5^ cells per insert. The medium was changed every other day, and TER was monitored regularly using a specialized cell ohmmeter (RE1600, Beijing KingGo Hi‐Tech, China). After the TER reached a stable plateau, the formation of a mature and functional barrier was confirmed by immunofluorescence staining of ZO‐1. To evaluate permeability, LA/DexP‐Cy5.5 nanoparticles at concentrations of 10, 40, and 80 µg/mL were added to the apical chamber. Fluorescein sodium salt and FITC‐dextran (40 kDa) were used as controls, respectively. At 0.5, 1, 2, and 4 h, 100 µL of medium was collected from the basolateral chamber and replaced immediately with an equal volume of fresh medium. Fluorescence intensity was measured using a microplate reader, and the relative permeability (%) was calculated as (fluorescence in basolateral chamber / initial fluorescence in apical chamber) × 100%. Barrier integrity was verified before and after each experiment by re‐measuring TER and performing ZO‐1 staining. Furthermore, LA/DexP or free DexP was applied apically, and basolateral samples were collected at the same time points. DexP concentration was quantified by HPLC. The Papp (cm/s) was calculated using the following equation: Papp = (dQ/dt) / (C_0_ × A), where dQ/dt is the amount of DexP permeated per second (µg/s), C_0_ is the initial drug concentration in the apical chamber (µg/mL), and A is the surface area of the Transwell membrane (0.33 cm^2^). For further validation in a stricter in vitro model, primary RPE cells were isolated from C57BL/6J mice. RPE purity was verified by RPE65 immunofluorescence staining. Cells were seeded and cultured on Transwell inserts under the same conditions, with TER monitoring until barrier stabilization. Barrier integrity was confirmed by ZO‐1 immunofluorescence staining. Permeability assays were then conducted following the same protocol as described above, including HPLC quantification of DexP in basolateral samples at 0.5, 1, 2, and 4 h. Papp values were calculated using the same equation.

### 4.15 Cellular Uptake and Thiol‐Mediated Uptake Mechanism

RAW264.7 cells were seeded in confocal dishes at 1×10^5^ cells/well. Upon adherence, the medium was replaced with fresh medium containing 80 µg/mL LA/DexP‐Cy5.5 and incubated for 1, 2, or 4 h. After incubation, cells were washed with PBS and stained with LysoTracker Red (50 nM, 30 min) for lysosomes and Hoechst 33342 (200 nM, 20 min) for nuclei. Imaging was performed using a confocal microscope (Leica SP8, Germany). The Cy5.5 fluorescence intensity and its colocalization with lysosomal signals were quantified using ImageJ software. To investigate the role of cell‐surface thiol groups in the uptake pathway, cells were pre‐treated with serum‐free medium containing 0.5 mM DTNB for 1 h. LA/DexP‐Cy5.5 (80 µg/mL) was then added directly, and the cells were incubated for an additional 4 h before processing for imaging as described above. Uptake fluorescence was compared to that of untreated controls.

### 4.16 Effect on cfDNA Internalization

For the internalization assay, RAW264.7 cells were treated either with FAM‐CpG (1 µM) alone or co‐treated with FAM‐CpG and LA/DexP‐Cy5.5 (80 µg/mL) for 1, 2, or 4 h. After incubation, cells were washed, stained with Hoechst 33342, and imaged by confocal microscopy to analyze the fluorescence intensity of internalized FAM‐CpG. To evaluate the cfDNA‐scavenging capacity of LA/DexP, cells were co‐incubated with unlabeled CpG (1 µM) and 80 µg/mL of LA/DexP, or with equivalent doses of LA or DexP (with DexP normalized to the amount contained in 80 µg/mL of LA/DexP) for 12 h. A PBS‐treated group served as the control. The culture supernatant was then collected, centrifuged at 3000 rpm for 10 min at 4°C, and the resulting supernatant was assessed for residual free DNA concentration using the dsDNA HS Assay Kit as previously described. For cytosolic DNA measurement, cells were harvested, fractionated using a cytoplasmic extraction kit (NE‐PER, Thermo Scientific), and DNA extracted from the cytoplasmic fractions was quantified using the same assay.

### 4.17 Detection of Intracellular ROS, Cellular Apoptosis, and BRB Integrity

RAW264.7 cells were stimulated with CpG (1 µM) for 12 h. After removal of the supernatant and washing to eliminate residual CpG, the cells were then treated with LA, DexP, or LA/DexP (with DexP dose equivalent to that contained in 80 µg/mL of LA/DexP) for an additional 12 h. A PBS‐treated group served as the control. After treatment, cells were incubated with 5 µM DCFH‐DA at 37°C for 20 min. Uninternalized probe was removed by washing three times with serum‐free medium. Nuclei were subsequently stained with Hoechst 33342. Finally, intracellular fluorescence was detected using an inverted fluorescence microscope (Leica DMi8, Germany) and quantified by flow cytometry. Apoptosis was analyzed by flow cytometry with an Annexin V‐FITC/PI detection kit and by fluorescence microscopy using a one‐step TUNEL assay kit, following the manufacturers’ protocols.

To assess BRB integrity, ARPE‐19 cells were cultured to form a confluent monolayer with established tight junctions, followed by the same treatment protocol as described for RAW264.7 cells. The expression and localization of tight junction proteins ZO‐1 and occludin were evaluated by immunofluorescence staining and Western blot analysis following standard procedures.

### 4.18 Immunofluorescence Staining

Samples (cells or tissue sections) were fixed with 4% paraformaldehyde for 10 min and blocked with 5% bovine serum albumin for 1 h at room temperature. They were then incubated with primary antibodies overnight at 4°C. After washing, the samples were incubated with fluorophore‐conjugated secondary antibodies for 1 h at room temperature in the dark. Nuclei were counterstained with DAPI, and images were acquired using a confocal microscope.

### 4.19 Western Blot Analysis

Following treatment, cells were washed three times with PBS and collected for subsequent analysis. Total protein was extracted from cells or retinal tissues using RIPA lysis buffer. Protein samples were separated by electrophoresis on a 4–20% gradient gel and transferred onto a PVDF membrane. The membrane was blocked with blocking buffer for 30 min at room temperature, followed by incubation with primary antibodies overnight at 4°C. After washing, the membrane was incubated with appropriate horseradish peroxidase (HRP)‐conjugated secondary antibodies for 1 h at room temperature. Protein bands were visualized using a Bio‐Rad gel imaging system and quantified with Image Lab software.

### 4.20 Quantitative Real‐Time Polymerase Chain Reaction (qRT‐PCR)

Total RNA isolation and subsequent cDNA synthesis were carried out with the SteadyPure Universal RNA Extraction Kit and the Evo M‐MLV RT Kit with gDNA Clean, respectively. Target gene amplification was conducted on a QuantStudio 5 system (Thermo Fisher Scientific) employing SYBR Green Master Mix. Relative mRNA expression was normalized to β‐actin and calculated using the 2−ΔΔCt method. All primer sequences utilized are provided in Table S1.

### 4.21 Elisa

Retinal tissues were collected from each group of mice and homogenized, followed by centrifugation to obtain the supernatant. Protein concentrations of COX‐2, iNOS, TNF‐α, IFN‐γ, IL‐17, IL‐23, TGF‐β, IL‐10, and Arg‐1 were measured using a commercial ELISA kit according to the manufacturer's protocol. Absorbance was read at 450 nm with a microplate reader, and the concentration of each factor was determined using a standard curve.

### 4.22 Flow Cytometric Analysis

To assess splenic T‐cell responses, murine spleens were aseptically harvested and mechanically dissociated. Splenocytes were then isolated by density‐gradient centrifugation using lymphocyte separation medium. The resulting mononuclear cells were either stimulated for 5 h with a cell activation cocktail containing brefeldin A (for Th1/Th17 profiling) or left unstimulated (for Treg analysis). Cells were subsequently stained for surface CD4, fixed, permeabilized, and incubated with fluorescently‐labeled antibodies against IFN‐γ, IL‐17, or Foxp3 (all from BioLegend). After washing and resuspension in PBS, samples were analyzed by flow cytometry. The frequencies of CD4^+^IFN‐γ^+^ (Th1), CD4^+^IL‐17^+^ (Th17), and CD4^+^CD25^+^Foxp3^+^ (Treg) subsets were quantified using FlowJo software.

### 4.23 In Vivo Distribution and Retinal Accumulation

LA/DexP‐Cy5.5 (2 mg/kg) or an equivalent dose of free Cy5.5 was administered intravenously to healthy mice (100 µL per mouse). Whole‐body fluorescence imaging was performed at designated time points using a small‐animal in vivo imaging system (IVScope 8200, Qinxiang Technology). Following imaging, mice were euthanized, and the heart, liver, spleen, lungs, kidneys, and eyes were excised for *ex vivo* fluorescence quantification. Eyeballs were embedded in OCT compound for preparation of retinal cryosections. After mounting with DAPI, the distribution of Cy5.5 signal across retinal layers was visualized by confocal microscopy.

For pharmacokinetic analysis, each group of mice received a single intravenous injection of LA/DexP or free DexP. At designated time points (0.5, 1, 2, 4, 6, 8, 24, and 48 h post‐injection), retinas were harvested and pooled from five mice into one sample (n = 3 per time point). Each pooled sample was weighed, homogenized in ice‐cold 80% acetonitrile containing 0.5% formic acid, and centrifuged (4°C, 12,000 rpm, 20 min). The supernatant was dried under nitrogen, reconstituted in ultrapure water, and analyzed by LC‐MS/MS. Chromatographic separation was performed on an Agilent Poroshell 120 EC‐C18 column (2.1 × 100 mm, 2.7 µm) at 40°C with a gradient of 0.1% formic acid in water and methanol at 0.30 mL/min. Mass spectrometry was conducted in positive ion mode with MRM monitoring of m/z 393.3 → 373.2 for DexP. Pharmacokinetic parameters were calculated using non‐compartmental analysis (Phoenix WinNonlin).

### 4.24 Biocompatibility Assessment

The systemic biocompatibility of LA/DexP was evaluated through in vitro hemolysis, in vivo hematological/biochemical profiling, and histopathological examination. For hemolysis assay, erythrocytes from C57BL/6 mice were incubated with LA/DexP (0–500 µg/mL). After centrifugation, supernatant absorbance was measured at 540 nm to calculate the hemolysis percentage. For in vivo assessment, healthy mice were randomly assigned to PBS, LA, DexP, or LA/DexP groups and received a tail‐vein injection (100 µL/mouse). After 14 days, blood was collected for complete blood count and serum biochemistry. Mice were then euthanized, and major organs (heart, liver, spleen, lung, kidney) were harvested, fixed in 4% paraformaldehyde, paraffin‐embedded, sectioned, and stained with H&E for histopathological evaluation.

### 4.25 Statistical Analysis

All data are presented as mean ± standard deviation. Statistical analyses were conducted using GraphPad Prism software. Differences between two groups were assessed by Student's t‐test. For comparisons among multiple groups, one‐way analysis of variance (ANOVA) was applied. If the assumptions of normality or homogeneity of variances were not met, the non‐parametric Kruskal–Wallis test was used instead. A *P*‐value < 0.05 was considered statistically significant.

## Author Contributions

Y. Wu was responsible for the experimental design, execution, data analysis, and drafting of the manuscript. W. Geng conceived the initial idea, acquired funding, conducted experiments, and revised the manuscript. Q. Dai conducted experiments and performed data analysis and curation. W. Zhang, P. Zhang, and Y. Lai contributed to sample preparation and animal experiments. C. Zhou, Y. Wang, Q. Cao, X. Luo, Y. Lai, and C. Huang assisted in sample processing and provided technical support. P. Yang acquired funding, conceived and supervised the study, and critically reviewed and revised the manuscript.

## Funding

This work was supported by the National Natural Science Foundation Key Program (82230032), the Joint Funds of the National Natural Science Foundation of China (U25A2090), the Chongqing Science and Technology Bureau Mountaineering Project (cyyy‐xkdfjh‐jcyj‐202301, cyyy‐xkdfjh‐cgzh‐202302), the Science and Technology Cooperation Project of the First Affiliated Hospital of Zhengzhou University (2025Hx39), the National Natural Science Foundation of China (3240100264), the China Postdoctoral Science Foundation (2024M753871), the Postdoctoral Fellowship Program of the China Postdoctoral Science Foundation (GZC20242140), and the Chongqing Medical Youth Top‐notch Talent Project (YXQN202583).

## Conflicts of Interest

The authors declare no conflicts of interest.

## Supporting information




**Supporting File**: advs76813‐sup‐0001‐SuppMat.docx.

## Data Availability

The data that supports the findings of this study are available in the supplementary material of this article.
