# Supplementary material for: An Aggregation‐Induced Polymerization Poly(Disulfide)‐Drug Nanoplatform for Autoimmune Uveitis Therapy via Inhibiting the cGAS‐STING Pathway
Source: Adv Sci (Weinh). 2026 Jul 29:e76813. Online ahead of print. doi: 10.1002/advs.76813 (PMC13418050; doi:10.1002/advs.76813)
Supplement: Supplementary file 1 — Supporting File: advs76813‐sup‐0001‐SuppMat.docx. [file ADVS-9999-e76813-s001.docx]

**Supplementary materials**

**An Aggregation-Induced Polymerization Poly(disulfide)-Drug Nanoplatform for Autoimmune Uveitis Therapy via Inhibiting the cGAS-STING Pathway**

Yuelan Wu, ^[a], 1^ Wenbo Geng, ^[a], 1^ Qinjin Dai, ^[a], 1^ Wanyun Zhang, ^[a]^ Yuxian Lai, ^[a]^ Pei Zhang, ^[a]^ Chunjiang Zhou, ^[a]^ Yinuo Wang, ^[a]^ Qingfeng Cao, ^[a]^ Xiang Luo, ^[a]^ Yujie Lai, ^[a]^ Changwei Huang, ^[a]^ Peizeng Yang * ^[a] [b]^

[a] Y. Wu, W. Geng, Q. Dai, W. Zhang, Y. Lai, P. Zhang, C. Zhou, Y. Wang, Q. Cao, X. Luo, Y. Lai, C. Huang, Prof. P. Yang

Ophthalmology Medical Center, The First Affiliated Hospital of Chongqing Medical University, Chongqing Key Laboratory for the Prevention and Treatment of Major Blinding Eye Diseases, Chongqing Branch (Municipality Division) of National Clinical Research Centre for Ocular Diseases, Chongqing, China

E-mail: [(peizengycmu@126.com)](mailto:(peizengycmu@126.com))

[b] Prof. P. Yang

Department of Ophthalmology, Joint Research Laboratory for Ocular Immunology and Retinal Injury Repair, The First Affiliated Hospital of Zhengzhou University, Henan International, Henan Province Eye Hospital, Zhengzhou, China

^1^ These authors contributed equally to this work.

* Corresponding author: Peizeng Yang


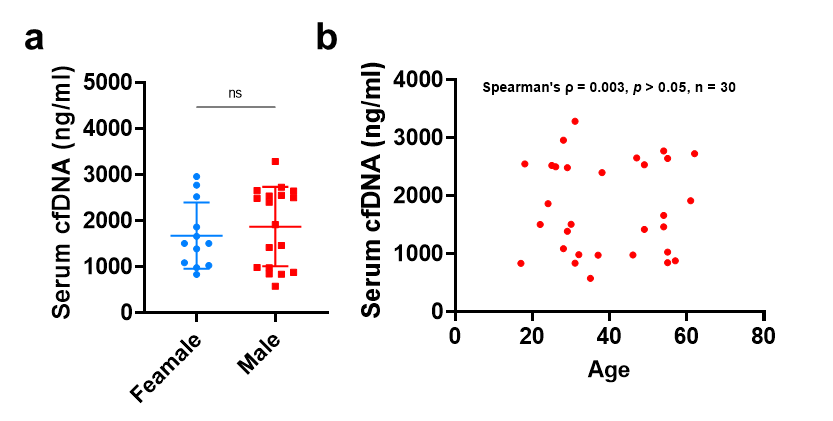


**Supplementary Figure S1.** Serum cfDNA levels are independent of age or sex in active AU patients. (a) Comparison of serum cfDNA levels between male and female patients with active AU. (b) Spearman’s rank correlation analysis between age and serum cfDNA levels (ρ = 0.003, *p* > 0.05, n = 30). Data are presented as mean ± SD.


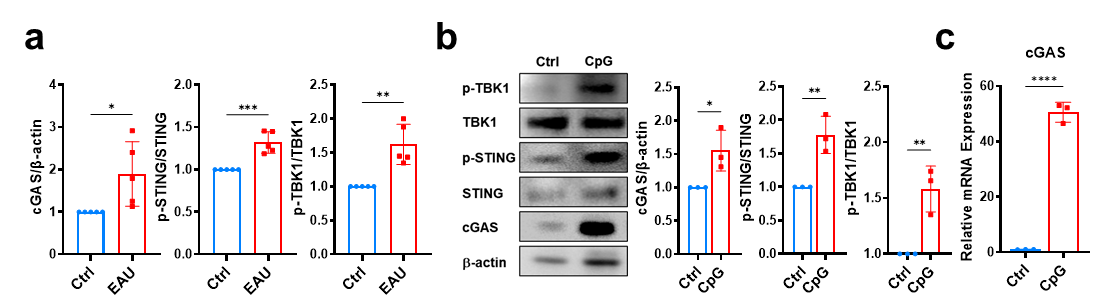


**Supplementary Figure S2.** cGAS-STING pathway protein and gene expression analysis. (a) Quantitative analysis of key cGAS-STING pathway proteins (cGAS/β-actin, p-STING/STING, p-TBK1/TBK1) in retinal tissues from normal controls and EAU mice (n = 5). (b) Representative Western blot images and quantitative analysis of key cGAS-STING pathway proteins in normal and CpG-stimulated macrophages (n = 3). (c) qRT-PCR analysis of cGAS mRNA expression in normal and CpG-stimulated macrophages (n = 3). Data are presented as mean ± SD. **p* < 0.05; ***p* < 0.01; ****p* < 0.001; *****p* < 0.0001.


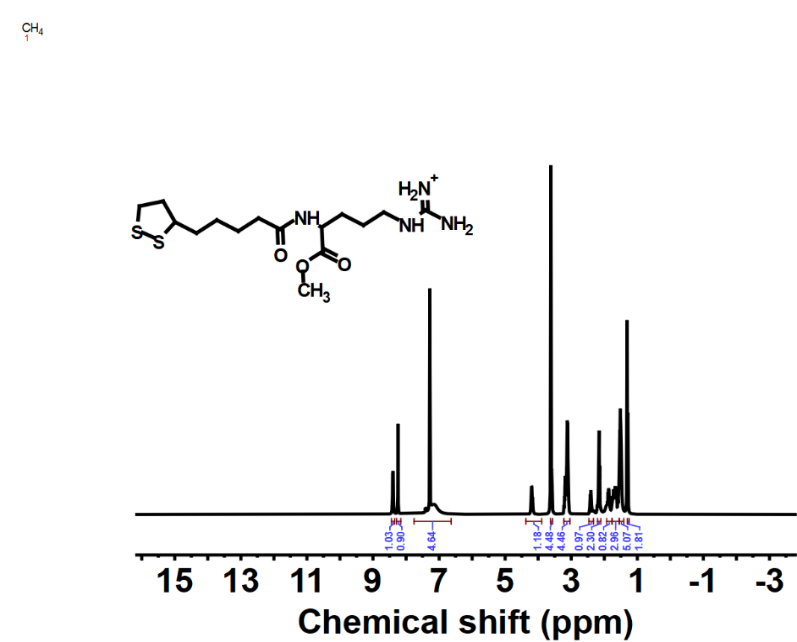


**Supplementary Figure S3**. ¹H NMR spectrum of LA in DMSO-d₆.





**Supplementary Figure S4.** MALDI-TOF MS spectrum of LA. The peak at *m/z* 377.17 corresponds to the protonated molecular ion [M+H]⁺.


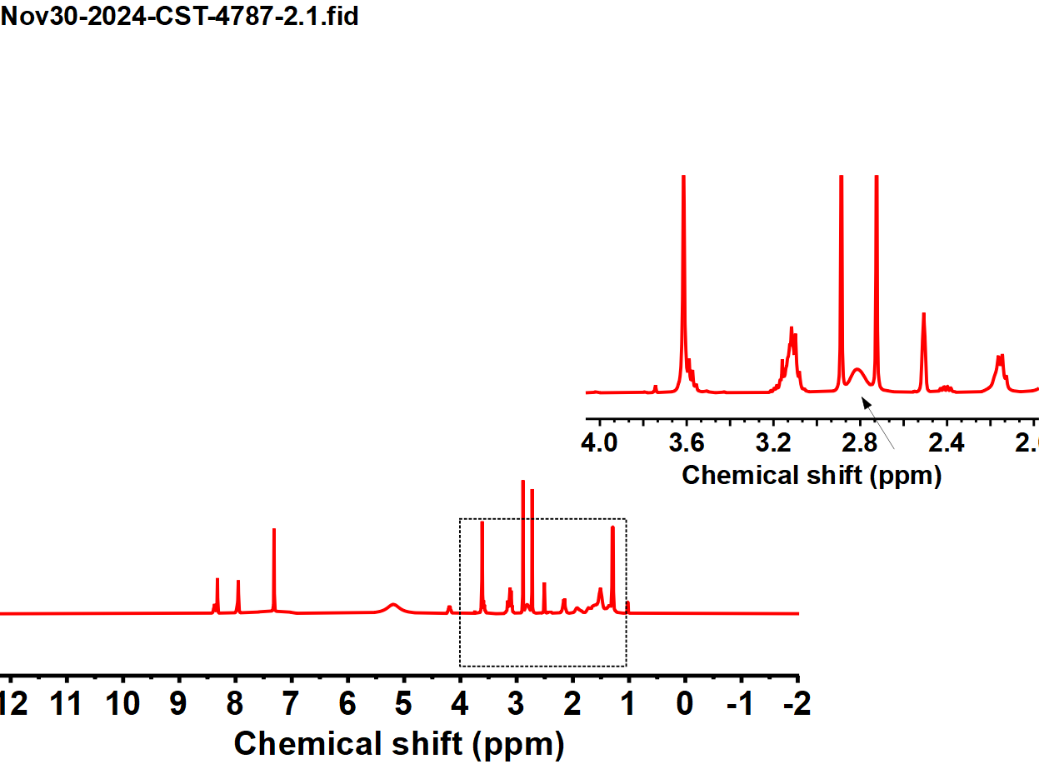


**Supplementary Figure S5**. ¹H NMR spectrum of LA/DexP in DMSO-d₆.





**Supplementary Figure S6**. MALDI-TOF MS spectrum of LA/DexP.


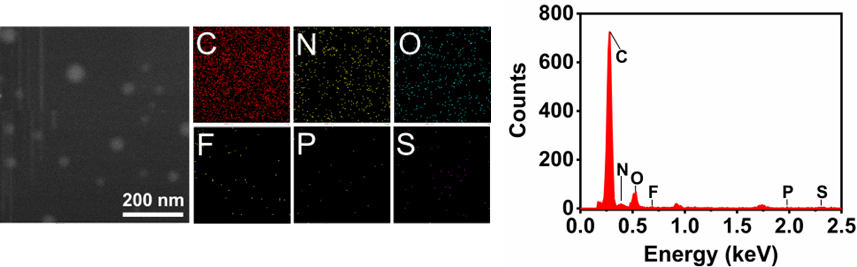


**Supplementary Figure S7.** TEM-EDS elemental mapping of LA/DexP. Scale bar: 200 nm.


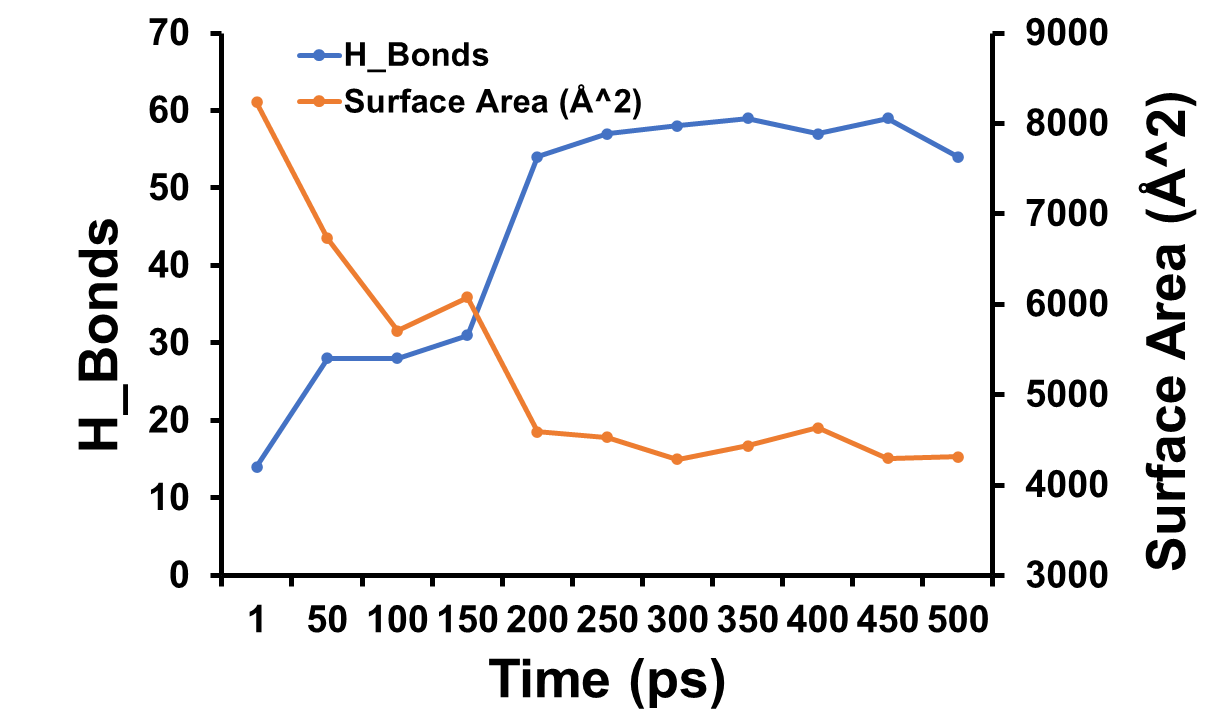


**Supplementary Figure S8.** Changes in solvent accessible surface area and hydrogen bond number during the self-assembly of LA/DexP nanoparticles.


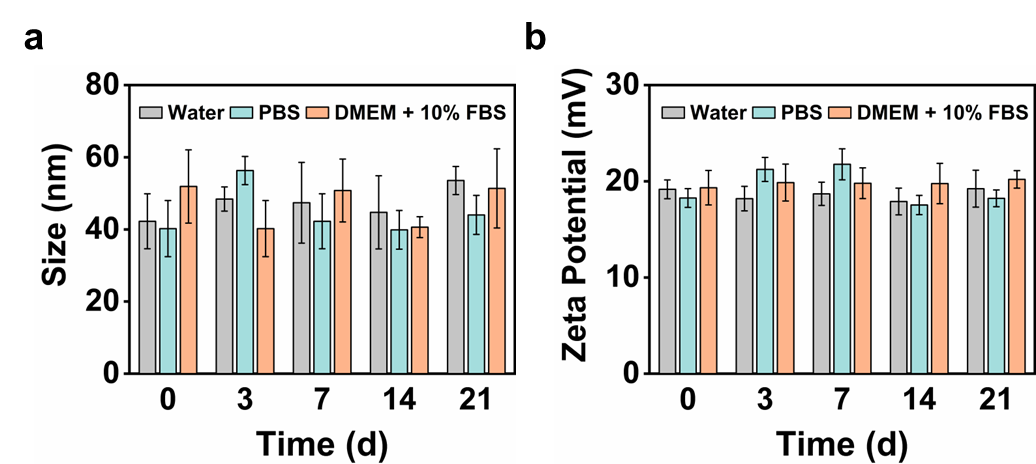


**Supplementary Figure S9.** (a) Size stability of LA/DexP incubated in water, PBS, and DMEM supplemented with 10% FBS at days 0, 3, 7, 14 and 21. (b) Zeta potential stability of LA/DexP measured at the same time points within the 21-day incubation period.


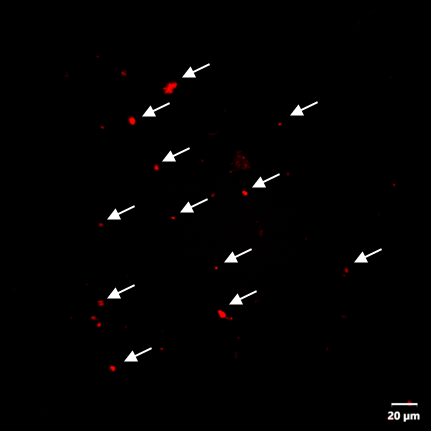


**Supplementary Figure S10**. Representative fluorescence image of LA/DexP-Cy5.5. Scale bar: 20 µm.


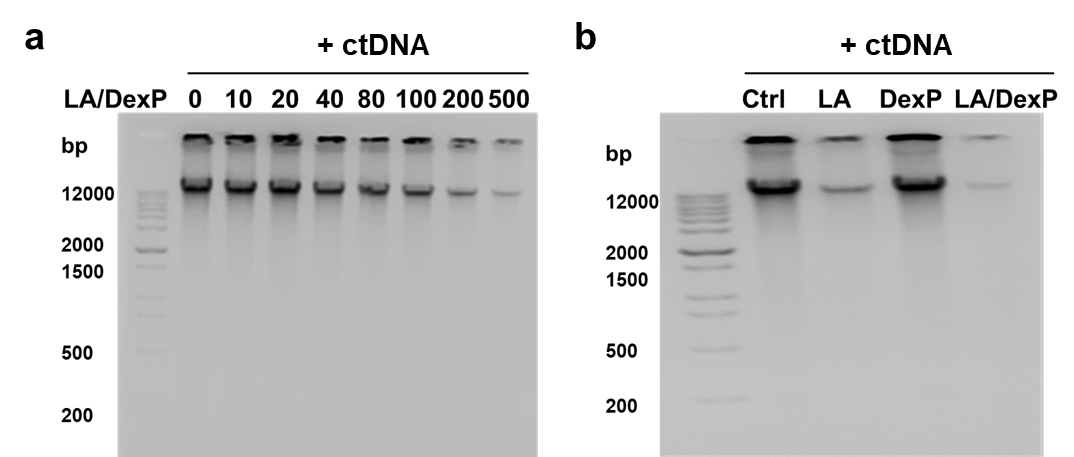


**Supplementary Figure S11.** Agarose gel electrophoresis analysis of ctDNA treated with various formulations. (a) ctDNA incubated with LA/DexP at concentrations of 0–500 μg/mL. (b) ctDNA incubated with PBS, LA, DexP, and LA/DexP.


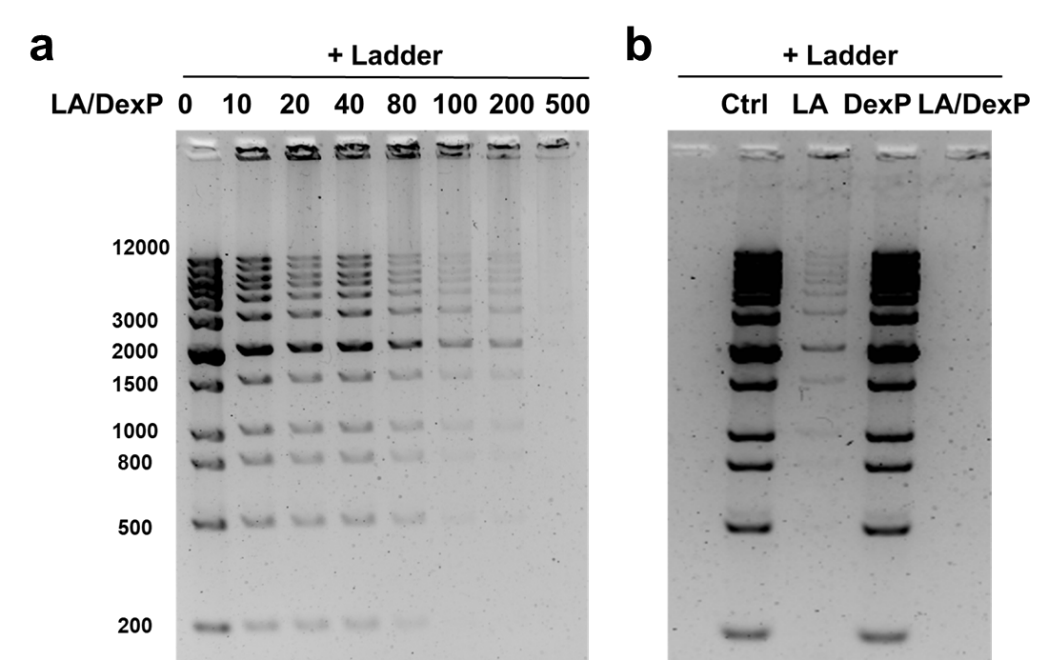


**Supplementary Figure S12.** Agarose gel electrophoresis analysis of DNA ladder treated with various formulations. (a) DNA ladder incubated with LA/DexP at concentrations of 0–500 μg/mL. (b) DNA ladder incubated with PBS, LA, DexP, and LA/DexP.


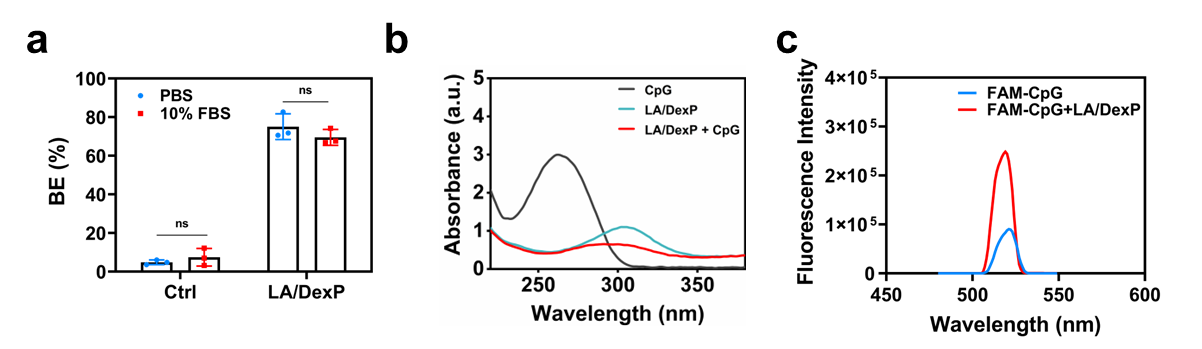


**Supplementary Figure S13.** Characterization of cfDNA scavenging by LA/DexP. (a) DNA scavenging efficiency of LA/DexP in PBS and in PBS containing 10% FBS. (b) UV‑Vis absorption spectra of CpG, LA/DexP, and the LA/DexP + CpG mixture. (c) Fluorescence intensity of FAM‑labeled CpG with and without LA/DexP.

**
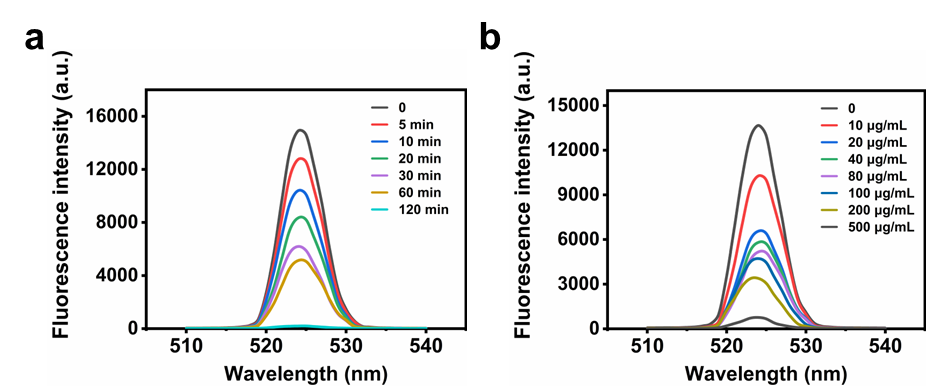
**

**Supplementary Figure S14.** Assessment of H₂O₂ scavenging by LA/DexP nanoparticles under varying incubation times and concentrations. (a) Time‑course measurement of H₂O₂ fluorescence intensity at a fixed LA/DexP concentration of 500 μg/mL. (b) Measurement of H₂O₂ fluorescence intensity with increasing concentrations of LA/DexP within a 60‑minute incubation period.


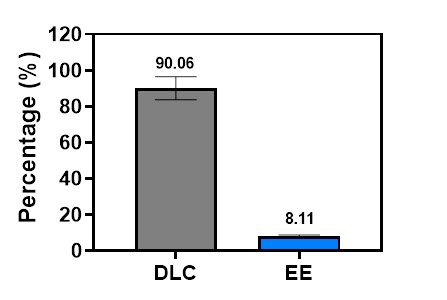


**Supplementary Figure S15.** Drug loading content (DLC) and encapsulation efficiency (EE) of LA/DexP.


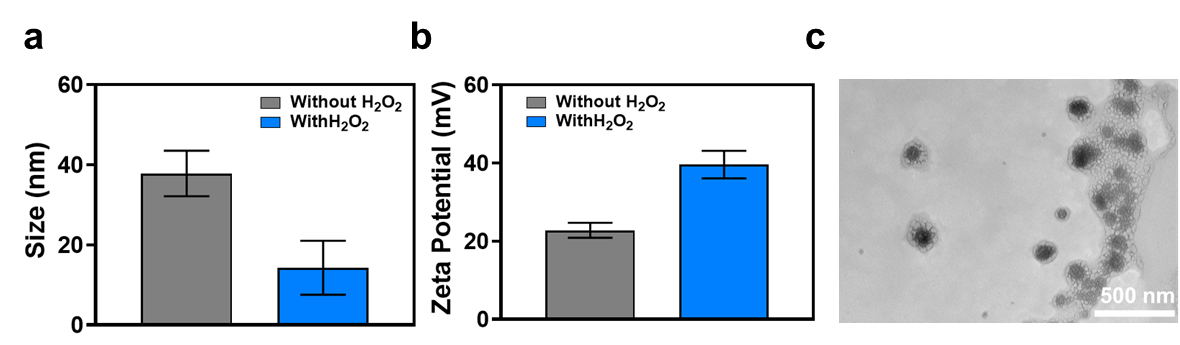


**Supplementary Figure S16.** Characterization of LA/DexP following H₂O₂ treatment. Hydrodynamic diameter (a) and zeta potential (b) of nanoparticles with or without 12 h H₂O₂ incubation. (c) Representative TEM images of LA/DexP after 12 h H₂O₂ treatment. Scale bar: 500 nm.


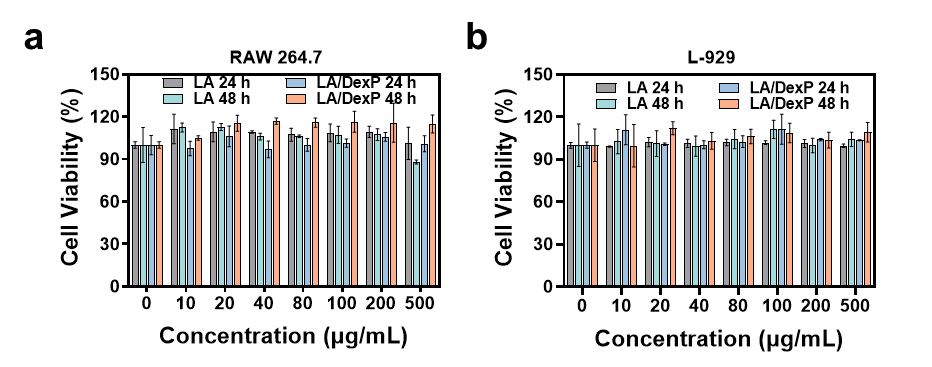


**Supplementary Figure S17.** Cell viability of RAW264.7 (a) and L‑929 (b) cells treated with LA or LA/DexP (0–500 μg/mL) for 24 and 48 h determined by the CCK‑8 assay.


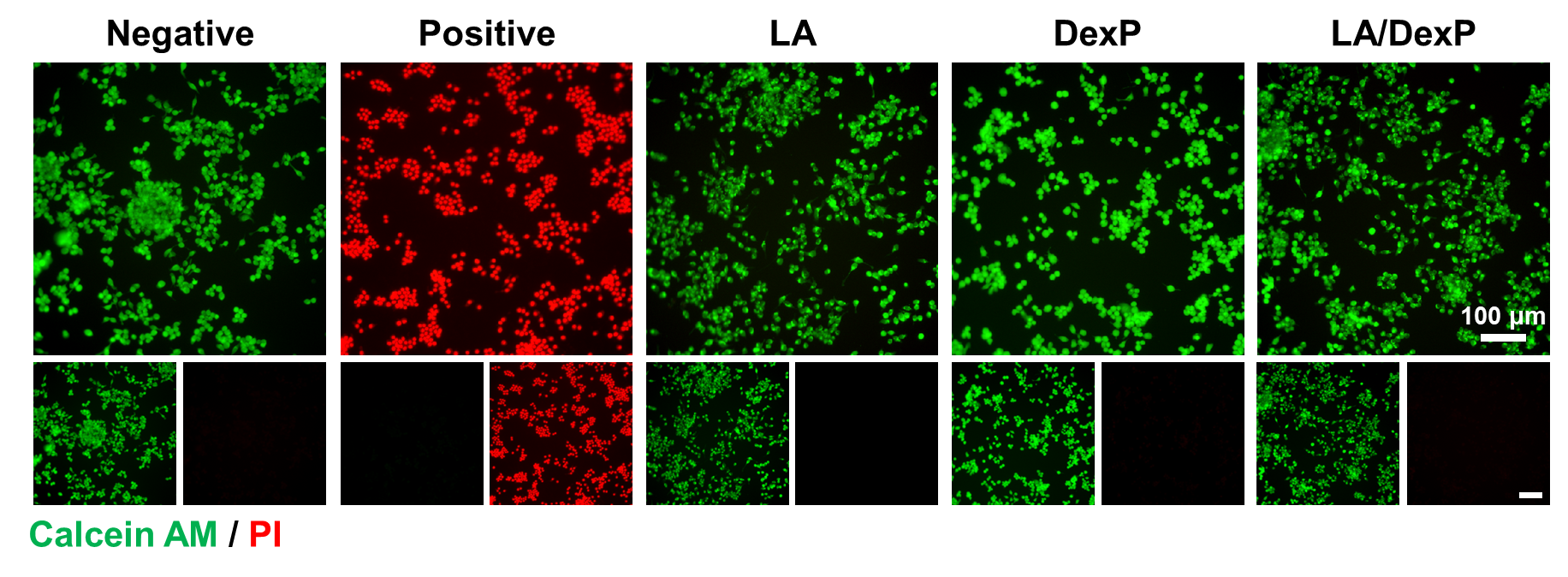


**Supplementary Figure S18.** Representative fluorescence micrographs of RAW264.7 cells after treatment with LA DexP or LA/DexP, stained with Calcein‑AM (green, live cells) and PI (red, dead cells). Scale bar: 100 µm.


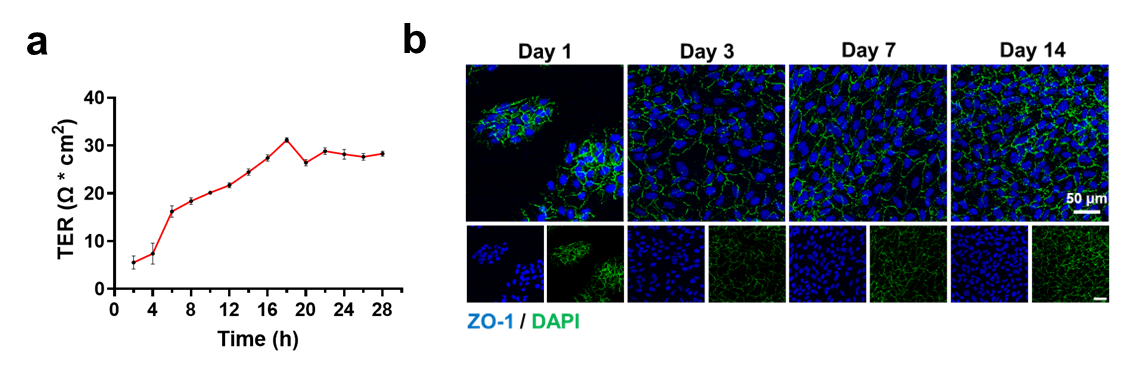


**Supplementary Figure S19.** Assessment of ARPE‑19 monolayer barrier integrity. (a) TER measurements recorded during the culture period. (b) Immunofluorescence staining for the tight‑junction protein ZO‑1. Scale bar: 50 µm.


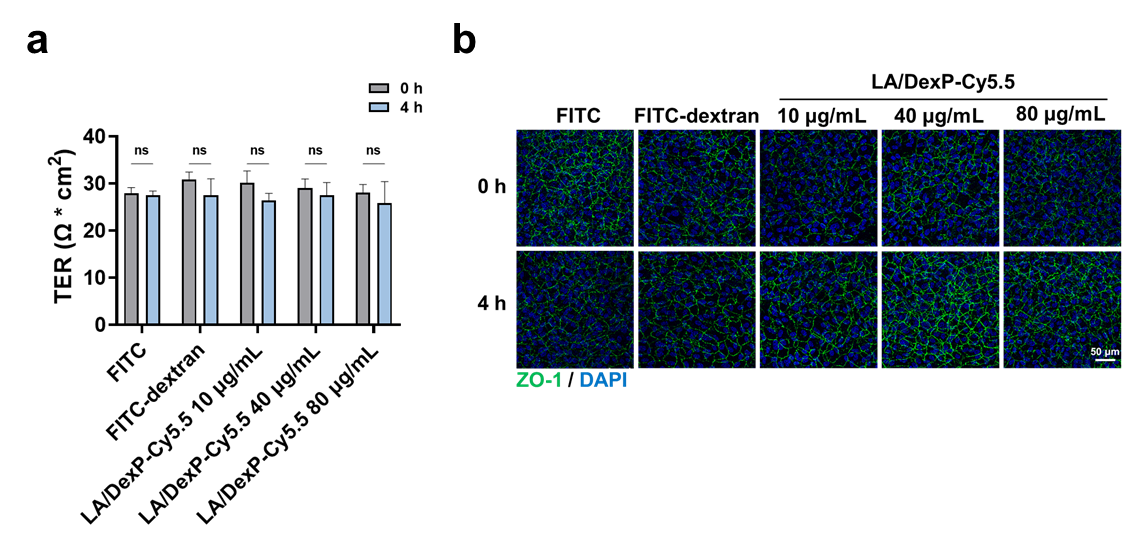


**Supplementary Figure S20.**  Evaluation of ARPE‑19 monolayer integrity. (a) TER measurements before (0 h) and after 4 h of treatment with 10–80 μg/mL LA/DexP-Cy5.5. (b) ZO‑1 immunofluorescence staining before (0 h) and after 4 h of treatment with 10–80 μg/mL LA/DexP-Cy5.5. Data are presented as mean ± SD (n = 3). Scale bar: 50 µm.

**
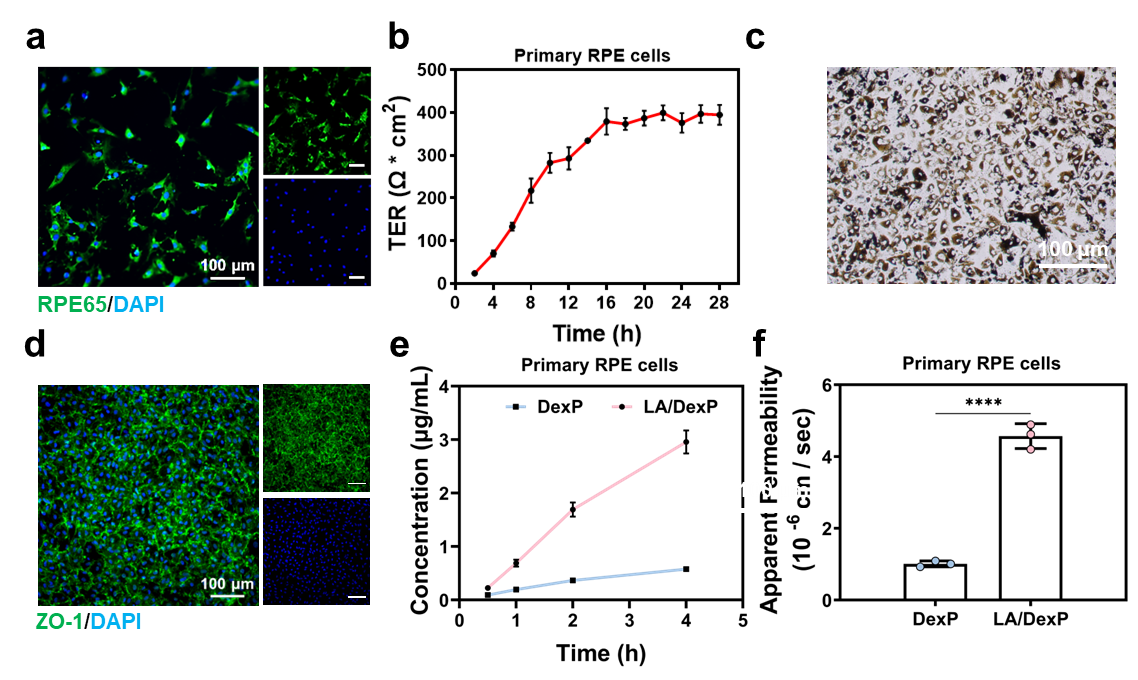
Supplementary Figure S21.** Validation of primary RPE barrier monolayers and barrier permeability of the LA/DexP nanoplatform. (a) Immunofluorescence staining for RPE65 in primary RPE cells cultured for 3 days post-isolation. Scale bar: 100 μm. (b) Time-dependent TER values of primary RPE monolayers over 28 days of culture. (c) Bright-field micrograph showing cobblestone morphology of mature primary RPE monolayer at day 20. Scale bar: 100 μm. (d) Immunofluorescence staining for ZO-1 in day-20 fully mature primary RPE monolayers. Scale bar: 100 μm. (e) HPLC quantification of DexP accumulation in the basolateral chamber at different time points following treatment with free DexP or LA/DexP across the RPE barrier. (f) Papp of free DexP and LA/DexP across primary RPE monolayers at 2 h post-treatment (n = 3). Data are presented as mean ± SD; *****p <* 0.0001.


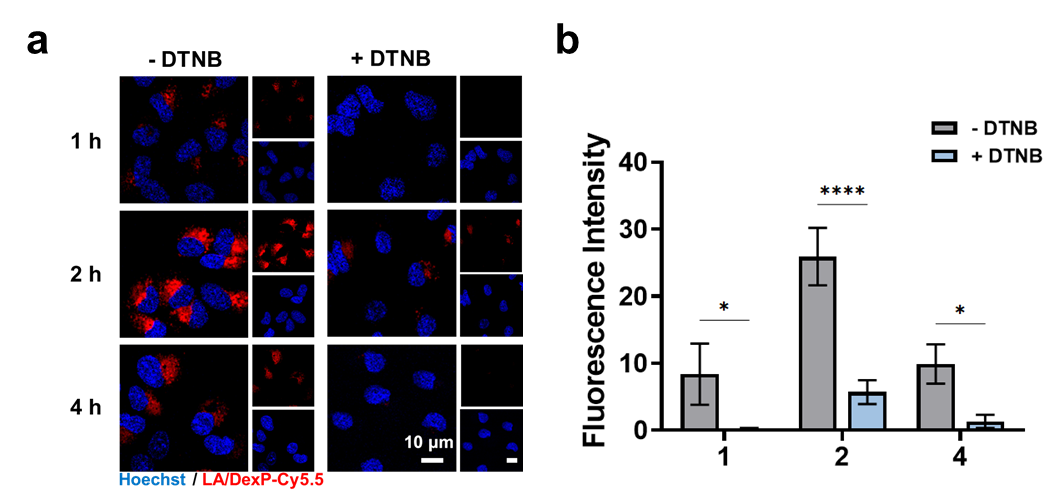


**Supplementary Figure S22.** Representative images (a) and quantitative analysis of fluorescence intensity (b) showing the time‑dependent cellular uptake of LA/DexP‑Cy5.5 in the presence or absence of DTNB pretreatment (n = 3). Scale bar: 10 µm.


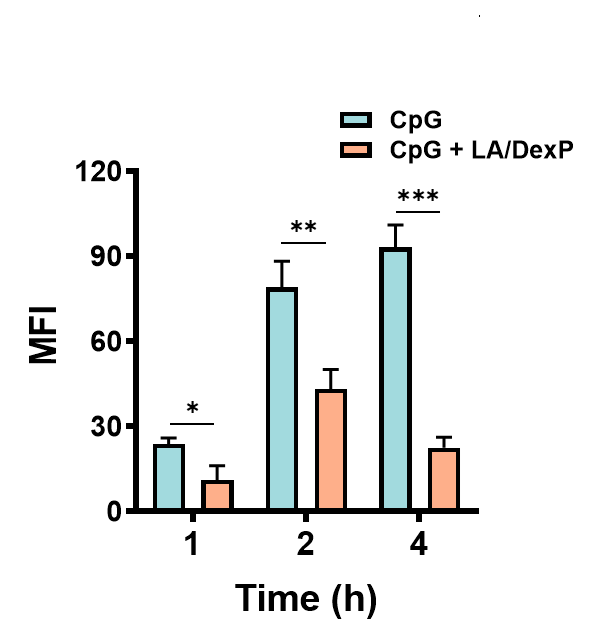


**Supplementary Figure S23.** Intracellular fluorescence intensity of CpG after incubation with CpG alone or CpG + LA/DexP-Cy5.5 for the indicated times (n = 3). Data are presented as mean ± SD; **p* < 0.05; ***p* < 0.01; ****p* < 0.001.


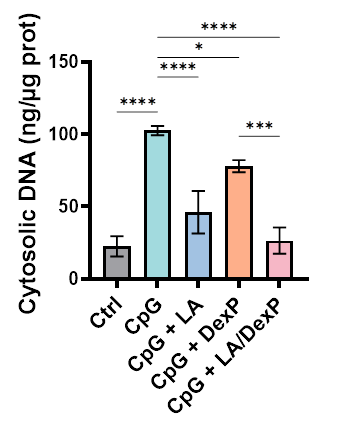


**Supplementary Figure S24.** Quantification of cytosolic DNA levels in RAW264.7 macrophages under different treatments (Ctrl, CpG, CpG + LA, CpG + DexP, CpG + LA/DexP, n = 3). Data are presented as mean ± SD; **p* < 0.05; ****p* < 0.001; *****p* < 0.0001.


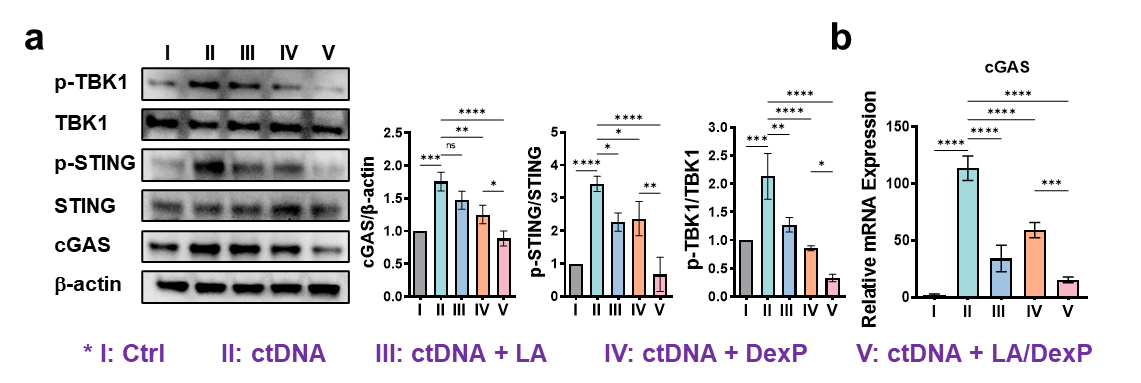


**Supplementary Figure S25.** LA/DexP inhibits the ctDNA-triggered cGAS-STING cascade in RAW264.7 macrophages. Cells were first stimulated with ctDNA, followed by treatment with LA, DexP, or LA/DexP. (a) Western blot analysis of cGAS, p-STING, and p-TBK1 protein expression. (b) qRT-PCR analysis of cGAS mRNA levels. Data are presented as mean ± SD (n = 3); **p* < 0.05; ***p* < 0.01; ****p* < 0.001; *****p* < 0.0001.


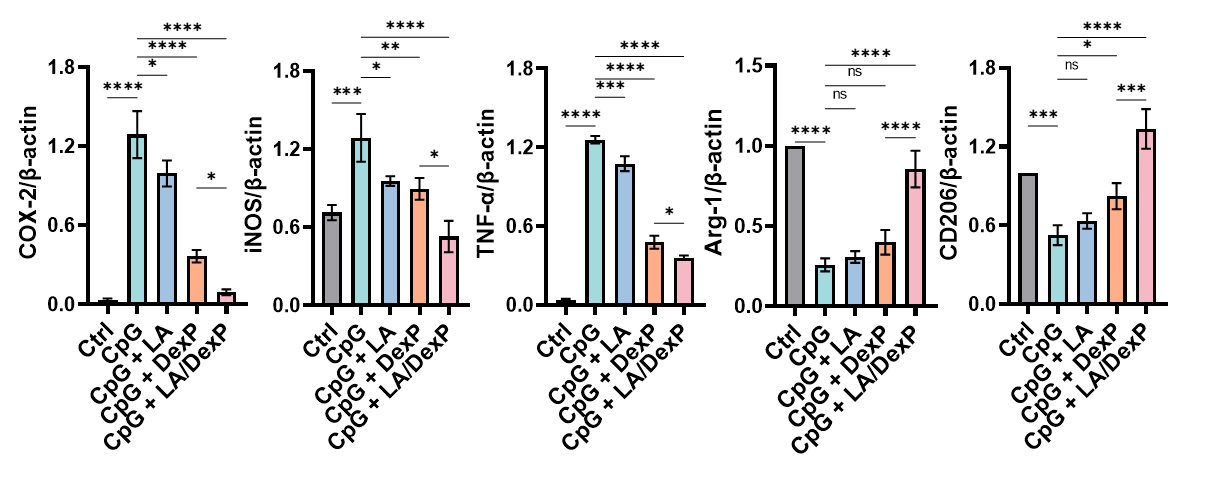


**Supplementary Figure S26.** Quantification of COX‑2, iNOS, TNF‑α, Arg‑1, and CD206 protein levels in RAW264.7 cells under different treatments (Ctrl, CpG, CpG + LA, CpG + DexP, CpG + LA/DexP) (n = 3). Data are presented as mean ± SD; **p* < 0.05; ***p* < 0.01; ****p* < 0.001; *****p* < 0.0001.


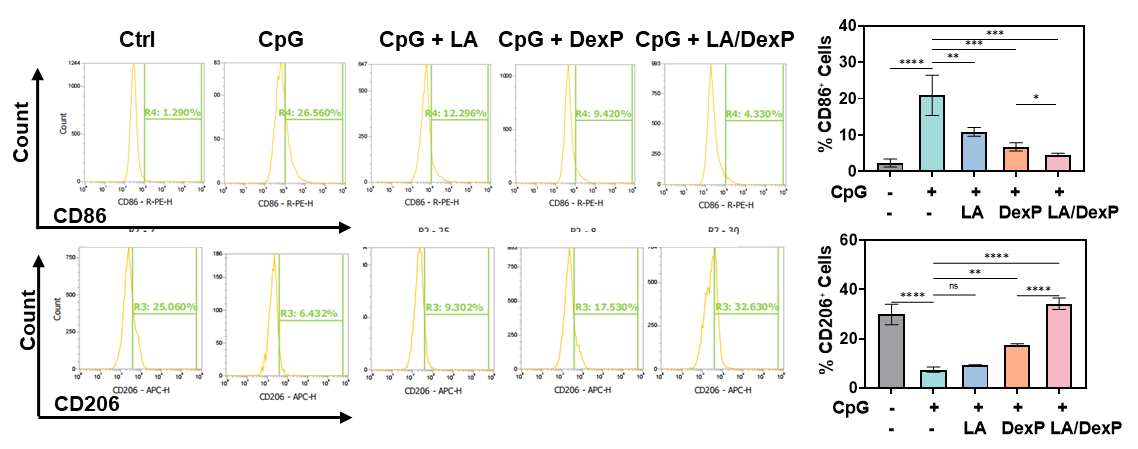


**Supplementary Figure S27.** Flow cytometric analysis of the proportions of M1 (CD86⁺) and M2 (CD206⁺) macrophages in RAW264.7 cells under different treatments (Ctrl, CpG, CpG + LA, CpG + DexP, CpG + LA/DexP) (n = 3). Data are presented as mean ± SD; **p <* 0.05; ***p <* 0.01; ****p* < 0.001; *****p* < 0.0001.


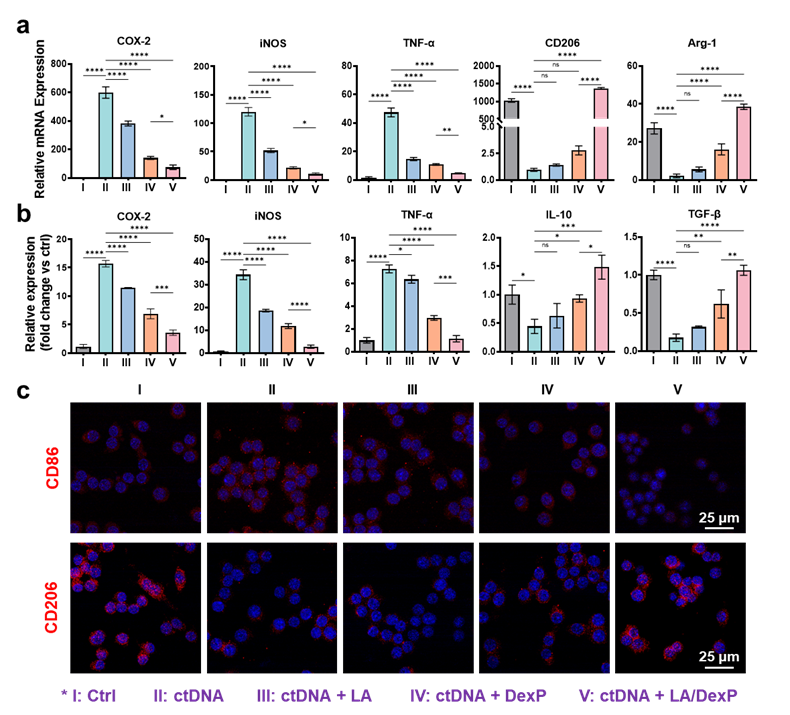


**Figure S28.** Macrophage inflammatory phenotypes after LA, DexP or LA/DexP treatment under ctDNA stimulation. (a) qRT-PCR analysis of pro-inflammatory (COX‑2, TNF‑α, iNOS) and anti-inflammatory (CD206, Arg-1) genes (n = 3). (b) ELISA analysis of secreted COX‑2, iNOS, TNF‑α, IL-10 and TGF-β in culture supernatants (n = 3). (c) Immunofluorescence staining of CD86 and CD206. Nuclei were stained with DAPI (blue). Scale bar: 25 μm. Data are presented as mean ± SD; **p* < 0.05; ***p* < 0.01; ****p* < 0.001; *****p* < 0.0001.


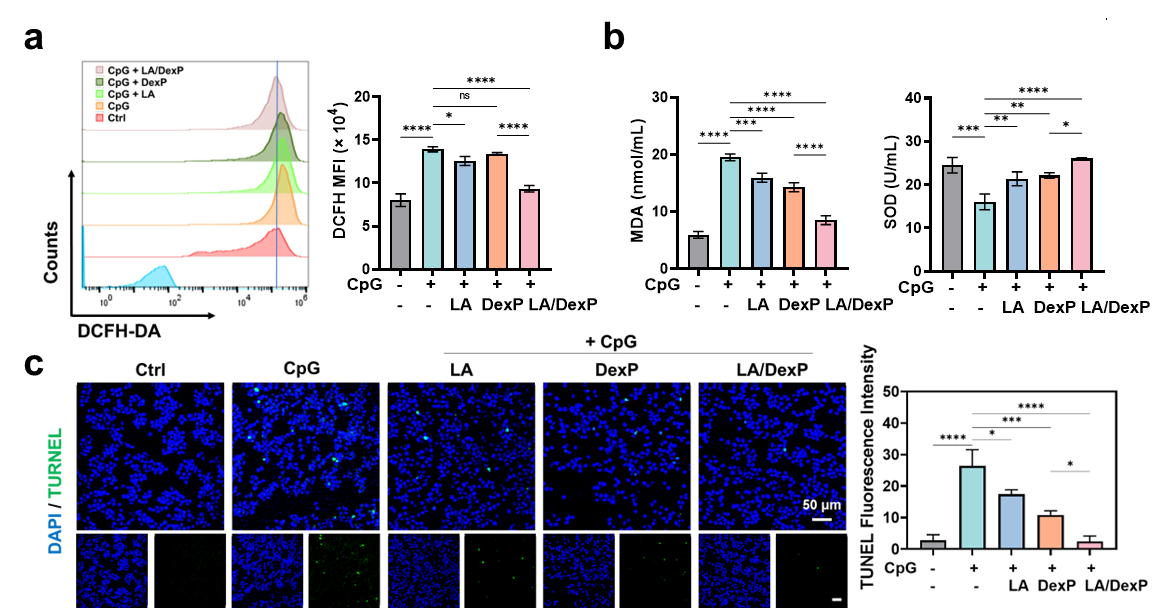


**Supplementary Figure S29.** Oxidative stress and apoptosis detection in RAW264.7 cells with different treatments (Ctrl, CpG, CpG + LA, CpG + DexP, CpG + LA/DexP). (a) Flow cytometric quantification of intracellular ROS levels. (b) Detection of MDA content and SOD activity. (c) Representative TUNEL staining images and quantitative statistics. Scale bar: 50 µm. Data are presented as mean ± SD (n = 3); **p* < 0.05; ***p* < 0.01; ****p* < 0.001; *****p* < 0.0001.


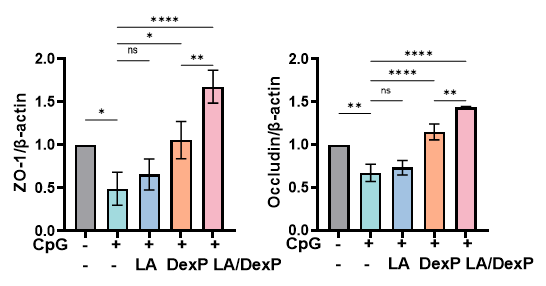


**Supplementary Figure S30.** Quantification of ZO-1 and occludin protein levels in ARPE-19 cells under different treatments (Ctrl, CpG, CpG + LA, CpG + DexP, CpG + LA/DexP) (n = 3). Data are presented as mean ± SD; **p* < 0.05; ***p* < 0.01; *****p* < 0.0001.


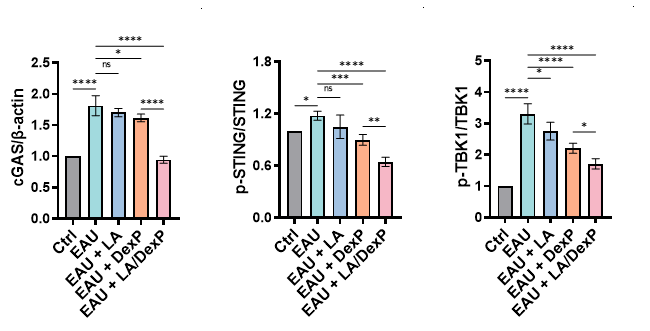


**Supplementary Figure S31.** Quantification of cGAS, p‑STING, and p‑TBK1 protein levels in retinal tissues under different treatments (Ctrl, EAU, EAU + LA, EAU + DexP, EAU + LA/DexP) (n = 4). Data are presented as mean ± SD; **p* < 0.05; ***p* < 0.01; ****p* < 0.001; *****p* < 0.0001.


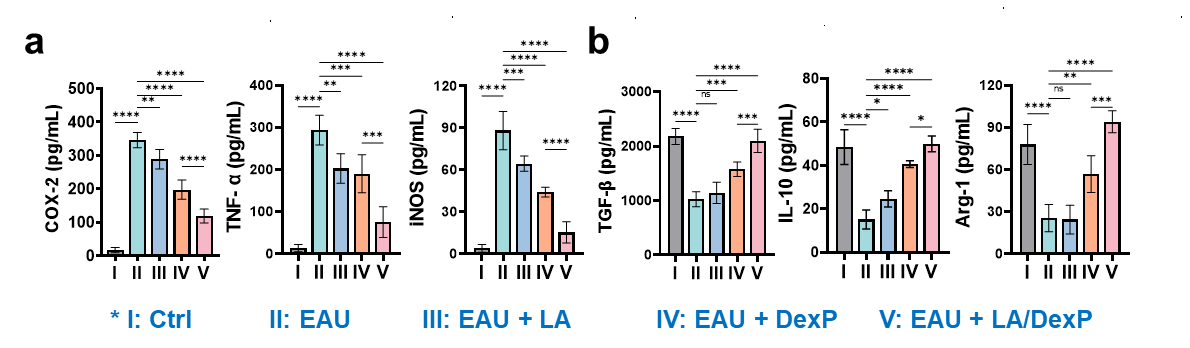


**Supplementary Figure S32.** Serum protein levels of inflammatory and anti‑inflammatory markers measured by ELISA in different treatment groups (Ctrl, EAU, EAU+LA, EAU+DexP, EAU+LA/DexP) (n = 5). (a) Levels of COX‑2, iNOS, and TNF‑α. (b) Levels of TGF‑β, IL‑10, and Arg‑1. Data are presented as mean ± SD; **p* < 0.05; ***p* < 0.01; ****p* < 0.001; *****p* < 0.0001.


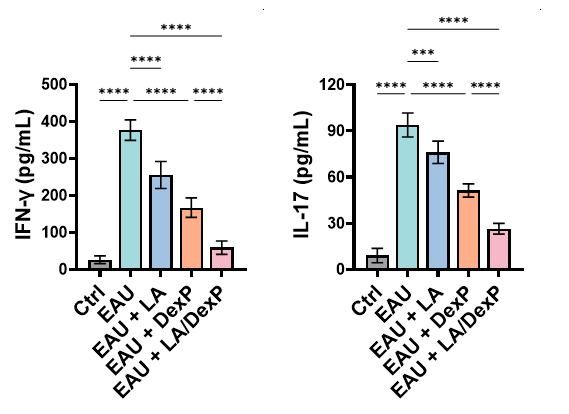


**Supplementary Figure S33.** Serum protein levels of IFN-γ and IL-17 measured by ELISA in different treatment groups (Ctrl, EAU, EAU+LA, EAU+DexP, EAU+LA/DexP) (n = 5). Data are presented as mean ± SD; ****p* < 0.001; *****p* < 0.0001.


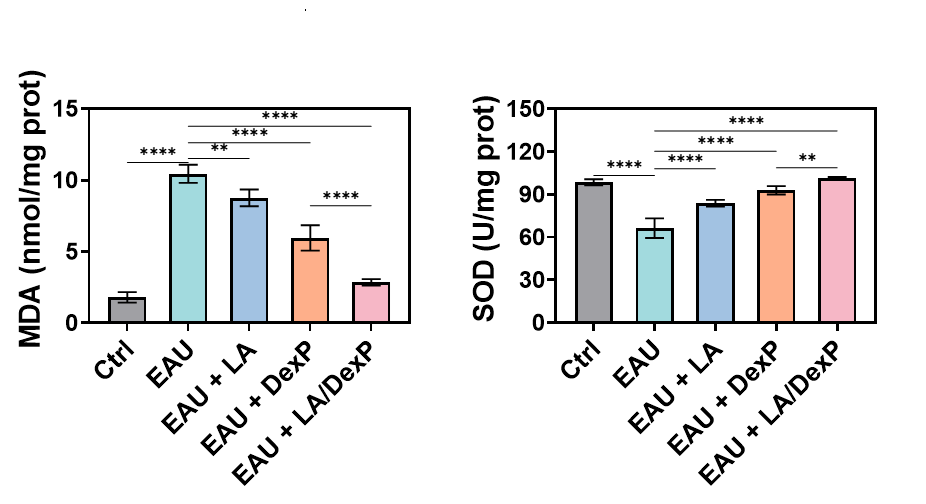


**Supplementary Figure S34.** MDA content and SOD activity in retinal tissues under different treatments (Ctrl, EAU, EAU+LA, EAU+DexP, EAU+LA/DexP). Data are presented as mean ± SD (n = 5); ***p* < 0.01; *****p* < 0.0001.

**Supplementary Figure S35.** Quantitative analysis of Evans blue extravasation in retinal tissues under different treatments (Ctrl, EAU, EAU+LA, EAU+DexP, EAU+LA/DexP). Data were normalized to the normal control group and were presented as mean ± SD (n = 4); ****p* < 0.001; *****p* < 0.0001.


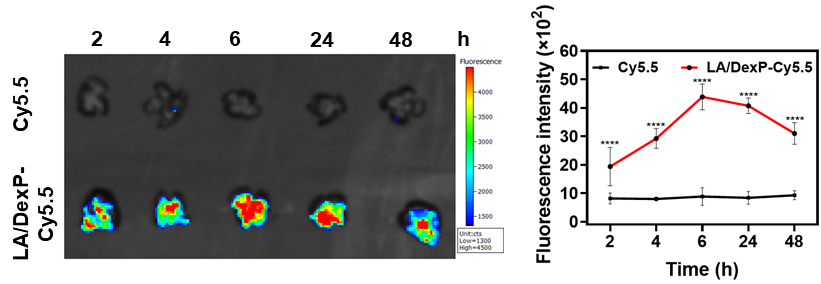


**Supplementary Figure S36.** *Ex vivo* fluorescence images of the retina at 2, 4, 6, 24, and 48 h post‑injection of free Cy5.5 or LA/DexP‑Cy5.5, with quantitative analysis of fluorescence intensity (n =5). Data are presented as mean ± SD. *****p* < 0.0001.

**

**Supplementary Figure S37.** Quantitative fluorescence intensity of the heart, liver, kidney, spleen, and lung harvested at 2, 4, 6, 24 and 48 h after intravenous injection of free Cy5.5 and LA/DexP-Cy5.5 (n = 5). Data are presented as mean ± SD; **p* < 0.05; ***p* < 0.01; ****p* < 0.001; *****p* < 0.0001.


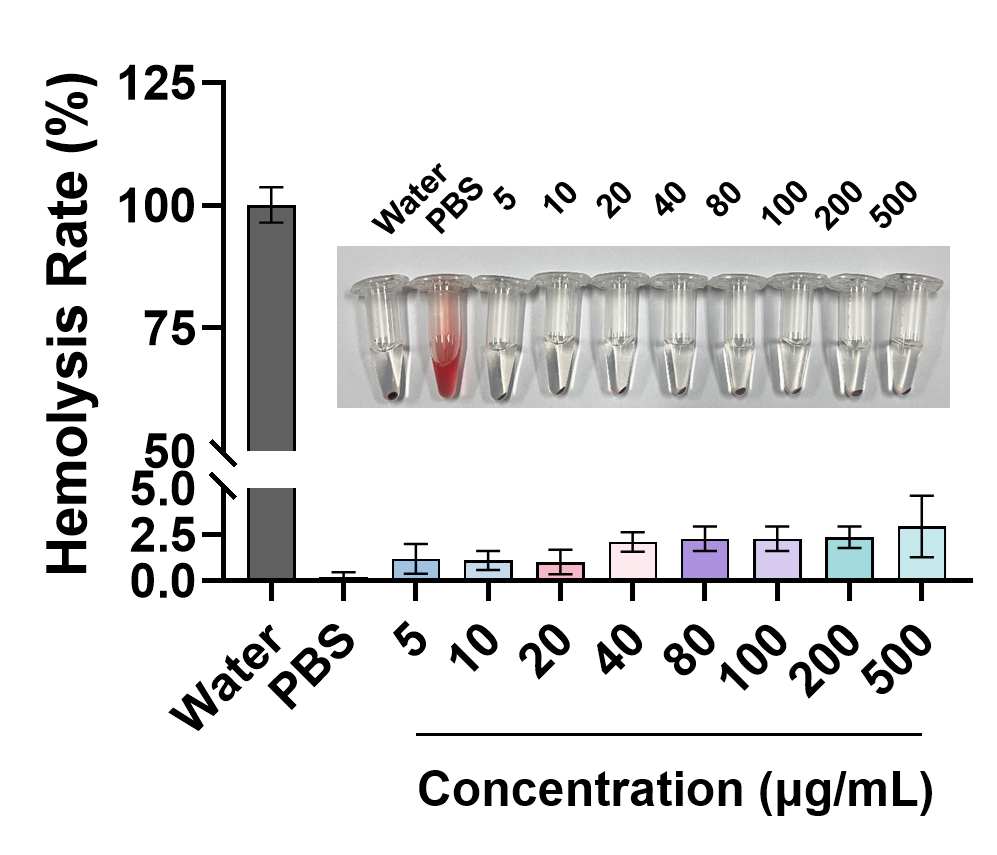


**Supplementary Figure S38.** Hemolysis rate of LA/DexP at different concentrations (n = 3). Data are presented as mean ± SD.


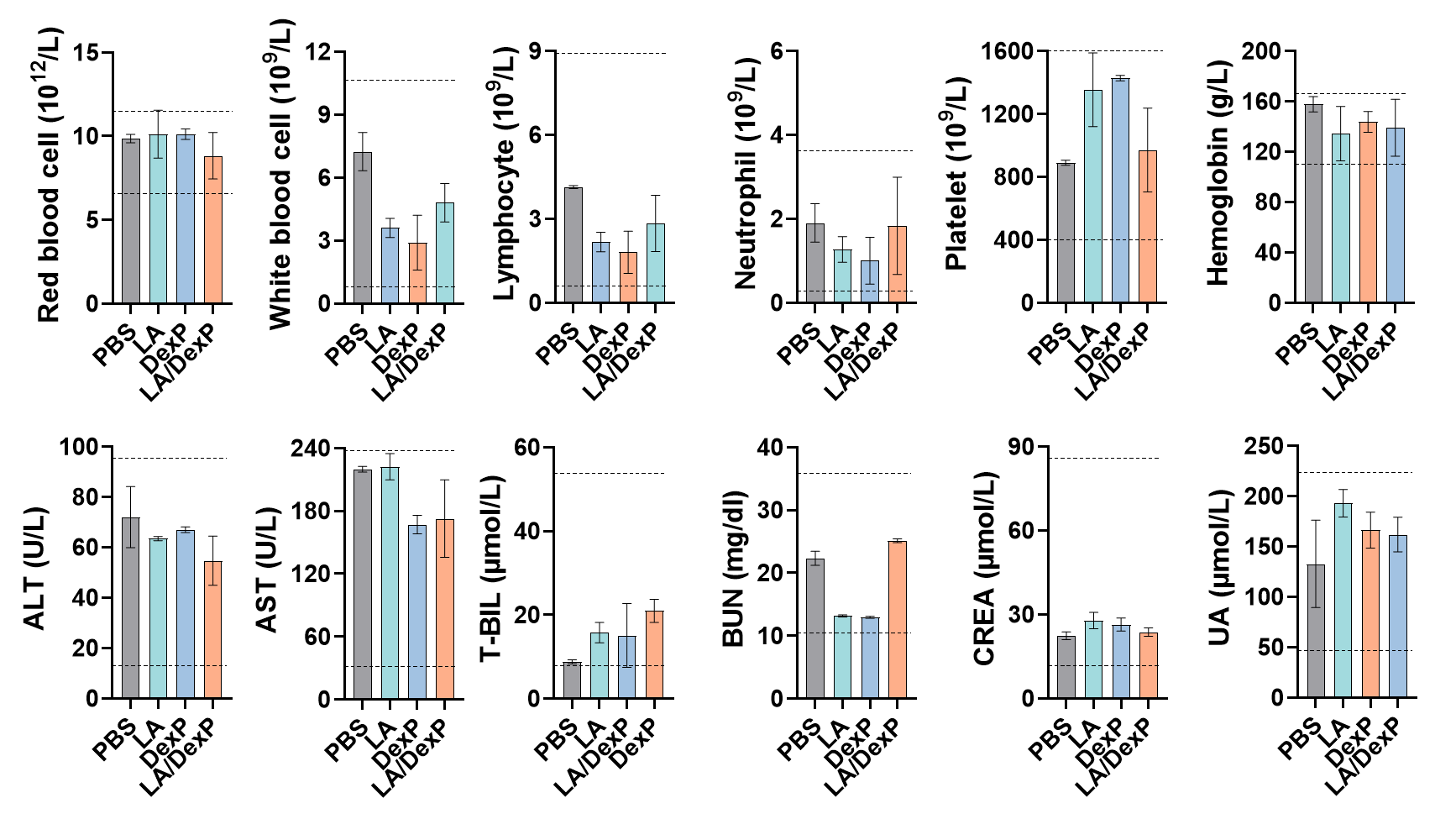


**Supplementary Figure S39.** Complete blood counts and serum levels of hepatic (ALT, AST, T‑BIL) and renal (BUN, CREA, UA) function markers from mice in different treatment groups (PBS, LA, DexP, LA/DexP) (n = 3). Data are presented as mean ± SD.


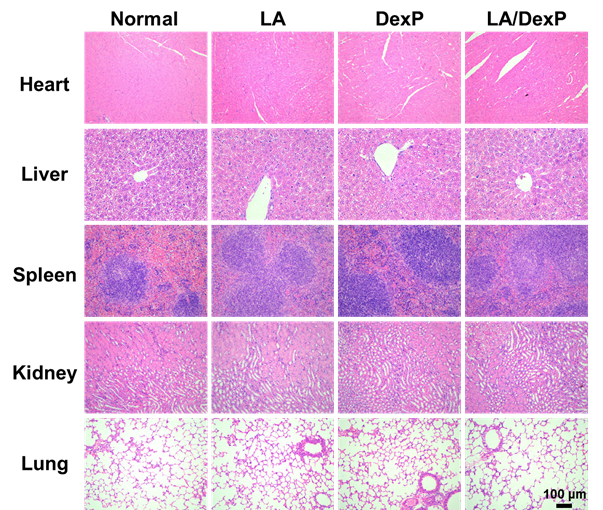


**Supplementary Figure S40.** H&E staining images of major organs (heart, liver, spleen, lungs, kidneys) from mice in different treatment groups (PBS, LA, DexP, LA/DexP) 14 days after injection.

**Supplementary Table S1.** Primer sequences used for qRT-PCR. β-actin was used as an endogenous reference gene for normalization.

| **Gene Name** | **Forward Primer (5’→3’)** | **Reverse Primer (5’→3’)** |
| --- | --- | --- |
| cGAS | AGCTTCCAGGAGGATGAGGA | GTGGCATCCAGATGCTGAAG |
| STING | AGAACCTGGCGGGAATCACC | TCCTTGGCGGAGGAAGGTCT |
| TBK1 | CCTGGAAGAAGGTGCTGAAAG | GCTCCAGTTGGTGGAAGTTCC |
| COX-2 | TGAGCAACTATTCCAAACCAGC | GCACGTAGTCTTCGATCACTATC |
| iNOS | GTTCTCAGCCCAACAATACAAGA | GTGGACGGGTCGATGTCAC |
| TNF-α | CTGAACTTCGGGGTGATCGG | GGCTTGTCACTCGAATTTTGAGA |
| Arg-1 | ATCAACACTCCGCTGACAACC | ATCTCGCAAGCCGATGTACAC |
| CD206 | CTCTGTTCAGCTATTGGACGC | TGGCACTCCCAAACATAATTTGA |
| β‑actin | GGCTGTATTCCCCTCCATCG | CCAGTTGGTAACAATGCCATGT |

**Supplementary Table S2.** Pharmacokinetic Parameters of DexP in Mouse Retina (Mean ± SD, n = 3).

| **Parameters** | **Free DexP** | **LA/DexP** | **Fold Increase** |
| --- | --- | --- | --- |
| T_max_ (h) | 1 | 6 | — |
| C_max_ (ng/mg) | 4.99 ± 0.27 | 8.29 ± 1.57 | 1.66 |
| Concentration at 6 h (ng/mg) | 2.16 ± 0.10 | 8.29 ± 1.57 | 3.84 |
| t_1/2_ (h) | 2.81 ± 0.24 | 11.09 ± 0.91 | 3.95 |
| AUC_0-∞_ (ng·h/mg) | 39.57 ± 3.80 | 119.76 ± 11.20 | 3.03 |
| MRT_0-∞_ (h) | 4.10 ± 0.38 | 15.90 ± 1.32 | 3.88 |
